# Hedgehog–BMP signalling establishes dorsoventral patterning in lateral plate mesoderm to trigger gonadogenesis in chicken embryos

**DOI:** 10.1038/ncomms12561

**Published:** 2016-08-25

**Authors:** Takashi Yoshino, Hidetaka Murai, Daisuke Saito

**Affiliations:** 1 Department of Zoology, Graduate School of Science, Kyoto University, Kitashirakawa, Sakyo-ku, Kyoto 606-8502, Japan; 2 Frontier Research Institute for Interdisciplinary Sciences (FRIS), Tohoku University, Aoba-ku, Sendai 980-8578, Japan

## Abstract

The gonad appears in the early embryo after several events: cells at the lateral plate mesoderm (LPM) undergo ingression, begin gonadal differentiation and then retain primordial germ cells (PGCs). Here we show that in the chicken embryo, these events are triggered on the basis of dorsoventral patterning at the medial LPM. Gonadal progenitor cells (GPCs) at the ventromedial LPM initiate gonadogenesis by undergoing ingression, whereas mesonephric capsule progenitor cells (MCPCs) at the dorsomedial LPM do not. These contrasting behaviours are caused by Hedgehog signalling, which is activated in GPCs but not in MCPCs. Inhibiting Hedgehog signalling prevents GPCs from forming gonadal structures and collecting PGCs. When activated by Hedgehog signalling, MCPCs form an ectopic gonad. This Hedgehog signalling is mediated by BMP4. These findings provide insight into embryonic patterning and gonadal initiation in the chicken embryo.

## Abstract (web summary)

Ingression of cells from the lateral plate mesoderm (LPM) initiates gonad differentiation but how these events are triggered is unclear. Here, the authors show that gonadal progenitor cells at the ventromedial LPM initiate gonadogenesis, and are activated by Hedgehog and BMP4 signalling.

## Graphical abstract

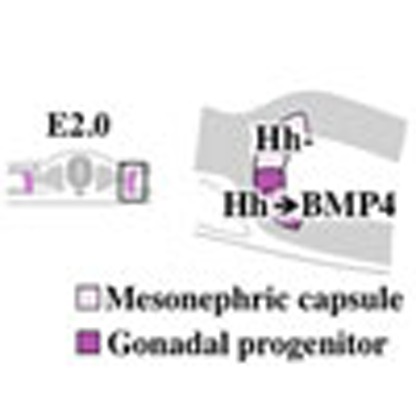

In animals, the gonads (testis and ovary) are essential for differentiation and maintenance of germ cells, which are required to create the progeny^1^,^2^. In the testis, somatic Sertoli cells support spermatogenesis through intimate interaction with germ cells^1^. Likewise, in the ovary, the oocyte develops through bidirectional signal exchanges with neighbouring somatic granulosa cells^2^. The testis and ovary secrete sex hormones to differentiate and maintain male and female characteristics, including the internal sex duct and other sexually dimorphic features^3^,^4^,^5^. Consequently, failure of gonadogenesis leads to infertility and disorders of sex differentiation^6^,^7^.

In vertebrates, gonadogenesis starts with the formation of sexually indifferent gonads, which emerge as genital ridges bilateral to the mesentery^8^,^9^. At early stages, the genital ridges do not exhibit any structural sexual dimorphisms, and maintain the capacity to differentiate into both testis and ovary. Subsequently, the bipotential gonad develops into either a testis or an ovary. The molecular mechanisms underlying sex determination (and subsequent sex differentiation of the gonad) have been investigated extensively in mice^3^,^10^,^11^. By contrast, the molecular mechanisms underlying the early stages of gonadal cell differentiation remain to be elucidated.

Bipotential gonads arise from a particular part of the coelomic epithelia^12^, which originate from the lateral plate mesoderm (LPM) in vertebrates^13^. The epithelia of the LPM undergo ingression to initiate genital ridge formation^14^,^15^. These cells start gonadal differentiation by expressing transcription factors, such as *Gata4*, *Lhx9*, *Wt1* and *Nr5a1* in mouse^16^,^17^,^18^. At nearly the same time, primordial germ cells (PGCs), which emerge in the extraembryonic region, migrate and localize to the genital ridge^19^,^20^ in all vertebrates. Accordingly, multiple processes must occur in parallel to accurately form the early stage of the gonad, including formation of the sexually bipotential genital ridge and acquisition of the capability to attract and retain PGCs. However, it remains unclear how these processes are triggered and orchestrated at the correct part of the vertebrate LPM.

Organogenesis is triggered based on early embryonic patterning. Hedgehog and bone morphogenetic protein (BMP) signals determine embryonic pattern formation and regulate various cellular behaviours, including migration and differentiation, in developing embryos^21^,^22^,^23^,^24^. In particular, Sonic hedgehog (SHH) and BMP4 play pivotal roles in the development of the LPM. In chicken embryo, LPM is formed based on mediolateral patterning of mesoderm, which is regulated by BMP4 (ref. 25). By contrast, in mouse and chicken embryo, gut mesenchymal cells derived from splanchnic mesoderm (ventral LPM) undergo orderly differentiation along the radial axis, established by SHH^26^,^27^. However, it remains unknown what kind of embryonic patterning is necessary for gonadogenesis, and how this embryonic patterning regulates the initiation of gonadogenesis in vertebrates.

In this study, we used *in ovo* electroporation to precisely localize the gonadal progenitor cells (GPCs) in a particular region of the LPM of day 2 chicken embryos (E2.0). Moreover, we found that Hedgehog and BMP4 signalling play crucial roles in the localization of GPCs by establishing a dorsoventral axis in the medial LPM, followed by the onset of gonadogenesis, in chicken embryo. Our results elucidate the molecular mechanisms that trigger gonad formation.

## Results

### Ventromedial LPM cells initiate gonadogenesis at E2.0

**Figure 1:**
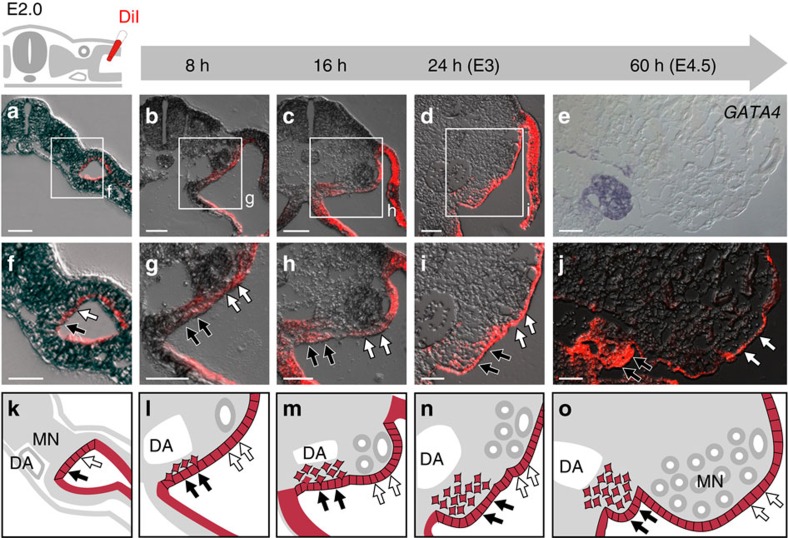
Localization of GPCs to a particular region of LPM in E2.0 chicken embryos. As illustrated at upper left, DiI was injected into a coelomic space enclosed to label the outer layer of LPM on the right side of chicken embryos at E2.0. (**a**) Transverse view of an embryo fixed immediately after DiI injection. (**b**–**d**) Transverse views of embryos incubated after DiI injection for the periods indicated at the top. (**e**) *GATA4* mRNA expression in the embryo at E4.5. (**f**–**i**) Magnified views of the boxed regions in **a**–**d**. Black and white arrows indicate GPCs and MCPCs, respectively. (**j**) DiI labelling in an embryo fixed 60 h after DiI injection (E4.5). (**k**–**o**) Illustrations of **f**–**j**. DiI-labelled cells are shown in red. DA, dorsal aorta; MN, mesonephros. Scale bars, 75 μm (**a**,**c**,**d**); 50 μm (**b**,**e**–**j**).

It is generally accepted that coelomic epithelial cells of the LPM undergo ingression to form the gonadal primordium^14^,^15^. In chicken embryos, such events should occur from E2.0 to E2.7. However, it remains unclear what subset of coelomic epithelial cells gives rise to the gonad primordium, as well as when these cells start migrating. To address these questions, we traced the lineage of LPM epithelial cells in E2.0 chicken embryos by labelling the cells with DiI (Fig. 1a,f, *n*=4; Fig. 1k). The ventromedial aspect of the labelled cells began to migrate soon after DiI injection (Fig. 1b,c,g,h, *n*=5; Fig. 1l,m), and obvious thickening was observed at the ventromedial aspect at E3.0 (Fig. 1d,i, *n*=5; Fig. 1n). These cells differentiated into gonadal cells, as revealed by the expression of its marker *GATA4* at E4.5 (Fig. 1e,j, *n*=4; Fig. 1o). These observations indicate that the cells at the ventromedial aspect of LPM are determined as the GPCs as early as E2.0 (Supplementary Fig. 1). This result is consistent with the idea that thickening of a particular region of the LPM in the E3.0 embryo is the first sign of gonadogenesis in the chicken^28^.

At E2.0, the ventromedial LPM cells formed an epithelial structure with a laminin-1-positive basement membrane and an atypical protein kinase C (PKC)-positive apical surface (Supplementary Fig. 2). These cells partially lost epithelial integrity at E3.0; the apical surface was maintained, but laminin-1 accumulated discontinuously (Supplementary Fig. 2). This degradation of epithelial integrity may be related to ingression of GPCs. The cells in the dorsomedial LPM covering the mesonephros (MCPCs; mesonephric capsule progenitor cells), which we previously termed Neph-CE^29^, were behaviourally distinct from GPCs at the ventral side. Unlike GPCs, the MCPCs did not undergo ingression, but remained as epithelia to form mesonephric capsule (Fig. 1, *n*=4; Supplementary Fig. 1) as we previously showed^29^. Subsequently, PGCs were attracted by the GPC-derived gonadal cells, but not by the MCPCs (Supplementary Fig. 3, *n*=5).

### Hedgehog signalling is activated in GPCs but not in MCPCs

On the basis of the observations described above, we assumed that the mechanisms that caused the differences between GPCs and MCPCs were crucial for triggering gonadogenesis in GPCs. SHH induces cell differentiation and migration in various contexts, including embryonic development and cancer metastasis^22^,^30^. *SHH* is expressed in endoderm adjacent to the GPCs, and the site of *SHH* expression is more distant from the MCPCs in both mouse and chicken embryos^31^,^32^; therefore, we assumed that the higher SHH concentration in GPCs relative to MCPCs is responsible for the differences in Hedgehog signalling activities and responses in GPCs and MCPCs.

**Figure 2:**
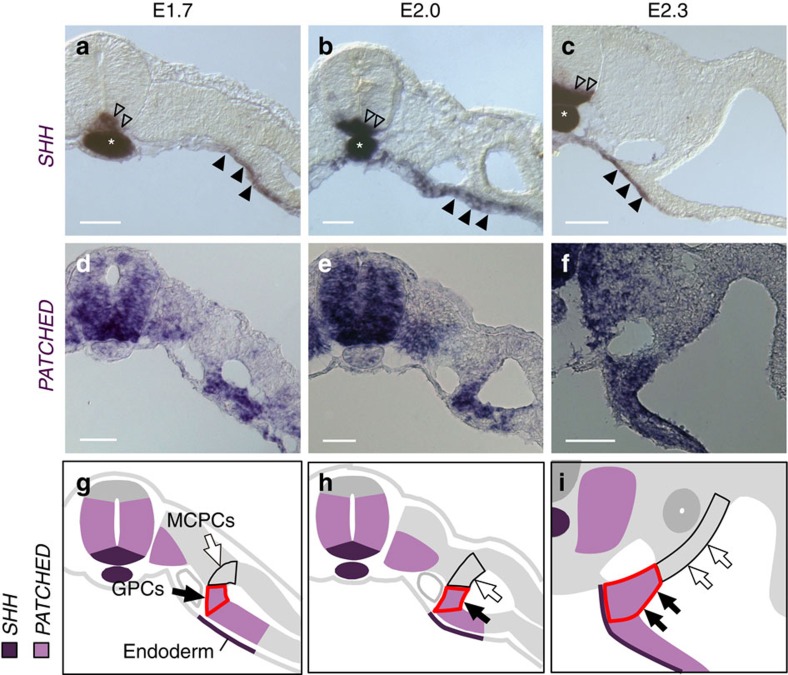
Activation of Hedgehog signalling in GPCs, but not MCPCs, before gonadogenesis. (**a**–**f**) mRNA expression of *SHH* (**a**–**c**) and its target gene *PATCHED* (**d**–**f**) are shown in chicken embryos at E1.7 (**a**,**d**), E2.0 (**b**,**e**) and E2.3 (**c**,**f**). SHH is expressed in the endoderm (black arrowheads) in addition to floor plate (open arrowheads) and notochord (asterisks). (**g**–**i**) Localizations of *SHH* and *PATCHED* mRNAs are illustrated with dark and light purple, respectively. *PATCHED* expression was detected in the future gonadal area (outlined in red) composed of coelomic epithelium (black arrows) and coelomic epithelial cell-derived underlying mesenchyme adjacent to the endoderm. *PATCHED* expression was not detected in MCPCs, which are more distant from the endoderm (white arrows). Scale bars, 50 μm.

To test this idea, we investigated the expression of a SHH downstream gene, *PATCHED,* in GPCs and MCPCs in E2.0 chicken embryos^33^,^34^. As reported previously, we detected *SHH* mRNA in the endoderm at E2.0, when GPCs begin to form gonadal primordium by undergoing ingression (Fig. 2a–c,g–i)^31^. Meanwhile, mRNA expression of *PATCHED* grew stronger in the GPCs. By contrast, expression of *PATCHED* was absent from the MCPCs (Fig. 2d–i).

SHH was expressed not only in the endoderm but also in axial structures (notochord and floor plate; Fig. 2a–c,g–i). However, even when the axial structures were removed, *PATCHED* expression was maintained in the GPCs (Supplementary Fig. 4, *n*=4). Furthermore, IHH (Indian Hedgehog), the other Hedgehog ligand in chicken embryos^35^, was not expressed in cells surrounding the GPCs (Supplementary Fig. 5). These results indicate that Hedgehog signalling is activated in the GPCs but not in the MCPCs of E2.0 chicken embryos, and that this differential activation pattern is most likely established by SHH secreted from the endoderm.

### Hedgehog signalling is required for GPCs to form gonad

**Figure 3:**
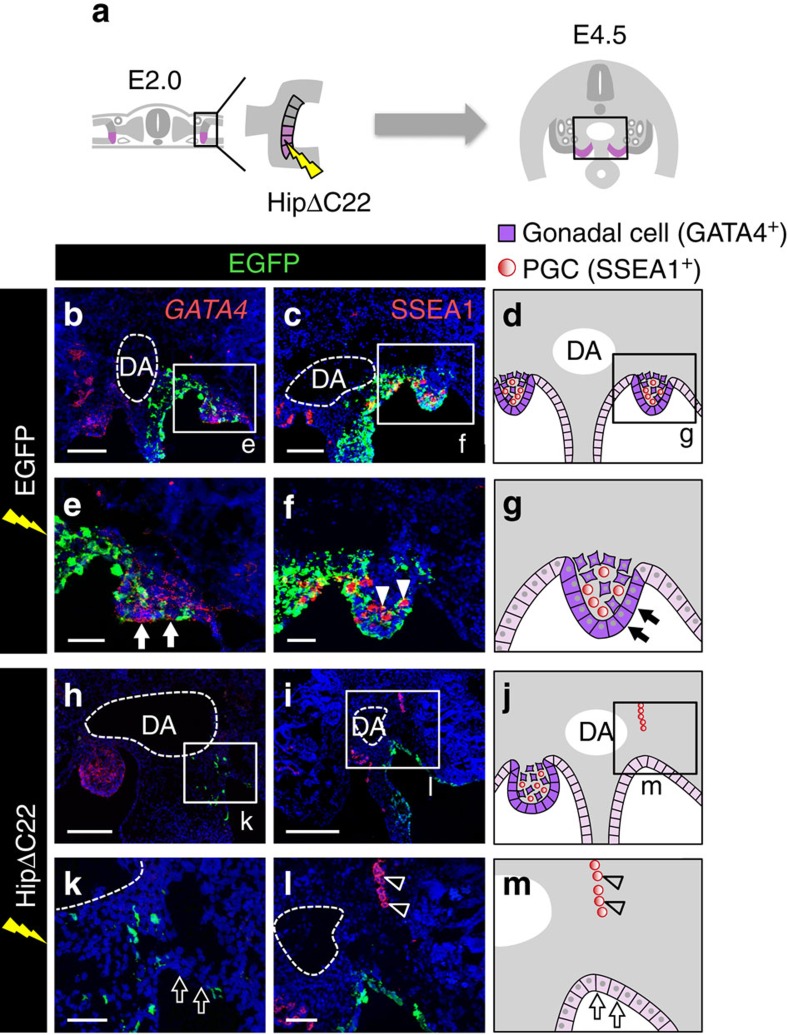
Hedgehog signalling is essential for gonadal development of GPCs. (**a**) Expression plasmid for HipΔC22, a secreted Hedgehog antagonist, was electroporated with EGFP expression plasmid into GPCs (pink) at E2.0. (**b**,**c**) Boxed region indicated in **a** in control EGFP-electroporated embryos (E4.5). *GATA4* mRNA (**b**) and SSEA1-positive primordial germ cells (PGCs) (**c**) were detected. (**d**) Illustrations of the development of gonads transfected with EGFP. (**e**–**g**) Magnified views of boxed regions in **b**–**d**. In control EGFP-electroporated embryos, *GATA4*-positive cells formed a swell (arrows in **e** and **g**) where SSEA1-positive PGCs localized (arrowheads in **f**). (**h**,**i**) Boxed region indicated in **a** in embryos (E4.5) transfected with HipΔC22 showing *GATA4* mRNA expression (**h**) and localization of PGCs (**i**). (**j**) Schematic representation of the events in **h** and **i**. (**k**–**m**) Magnified views of boxed regions in **h**–**j**. In HipΔC22-overexpressing embryos, *GATA4* was not expressed in the presumptive gonadal cells, the genital ridge was not formed (arrows in **k** and **m**), and the PGCs scattered (arrowheads in **l** and **m**). DA, dorsal aorta. Scale bars, 100 μm (**b**,**c**,**h**,**i**); 50 μm (**e**,**f**,**k**,**l**).

Next, we examined how differential Hedgehog signalling activity induces differential behaviours of GPCs and MCPCs. First, we inhibited Hedgehog signalling to investigate whether GPCs required the signal to form gonadal primordium. Because impairment of Hedgehog signalling throughout the body causes embryonic lethality before the formation of gonadal primordium^32^, we blocked Hedgehog signalling in a spatiotemporally controlled manner. We performed region-specific gene manipulation by a modification of our previously established method for electroporation of genes into MCPCs^29^. Specifically, we adjusted the position of the electrode slightly to direct the electric pulse ventrally into E2.0 embryos so that the genes were transferred mainly into GPCs (Fig. 3a).

Hedgehog-interacting protein (Hip) lacking the last 22 C-terminal amino-acid residues (HipΔC22; Fig. 3a) competitively inhibits binding of Hedgehog ligand to its receptor in the extracellular space^34^,^36^. Forced expression of HipΔC22 in GPCs led to the downregulation of Hedgehog signalling specifically in these cells 12 h after electroporation (Supplementary Fig. 6, *n*=6). In enhanced green fluorescent protein (EGFP)-electroporated (control) embryos, *GATA4*-expressing gonadal primordium started to form ridge structures bilateral to the mesentery at E4.5 (Fig. 3b,e, *n*=10; Fig. 3d,g). By contrast, when Hedgehog signalling was interrupted by HipΔC22 at E2.0, no genital ridge was formed at the presumptive gonadal area on the electroporated side. Consistent with this, cells in this region failed to express *GATA4* (Fig. 3h,k, *n*=5; Fig. 3j,m). Moreover, when HipΔC22 was overexpressed in GPCs at E3.0, they formed a GATA4+ genital ridge (Supplementary Fig. 7, *n*=6). These results indicate that Hedgehog signalling activated in GPCs from E2.0 to E3.0 causes them to differentiate into gonadal cells and form a genital ridge.

To investigate whether Hedgehog signalling endows the gonadal primordium with the ability to attract and retain PGCs, we examined the effect of HipΔC22 overexpression on the localization of PGCs marked by the expression of SSEA1 or CVH. In chicken embryos, PGCs floating in the blood flow migrate almost equally to the left and right gonad through prospective mesentery (Supplementary Fig. 3, *n*=4; Fig. 3c,f, *n*=10; Fig. 3d,g; Supplementary Fig. 8)^19^,^37^. However, when Hedgehog signalling was inhibited as described above, the PGCs localized ectopically; moreover, they were less abundant in the gonad at electroporated side and more abundant on the untreated side (Fig. 3i,l, *n*=5; Fig. 3j,m; Supplementary Fig. 8), possibly because the gonads did not attract or retain PGCs on the electroporated side and more PGCs were attracted to the gonad on the untreated side. Thus, Hedgehog signalling is indispensable for the settlement of PGCs during the early stage of gonadal development.

### SHH is sufficient to trigger gonadogenesis in MCPCs

**Figure 4:**
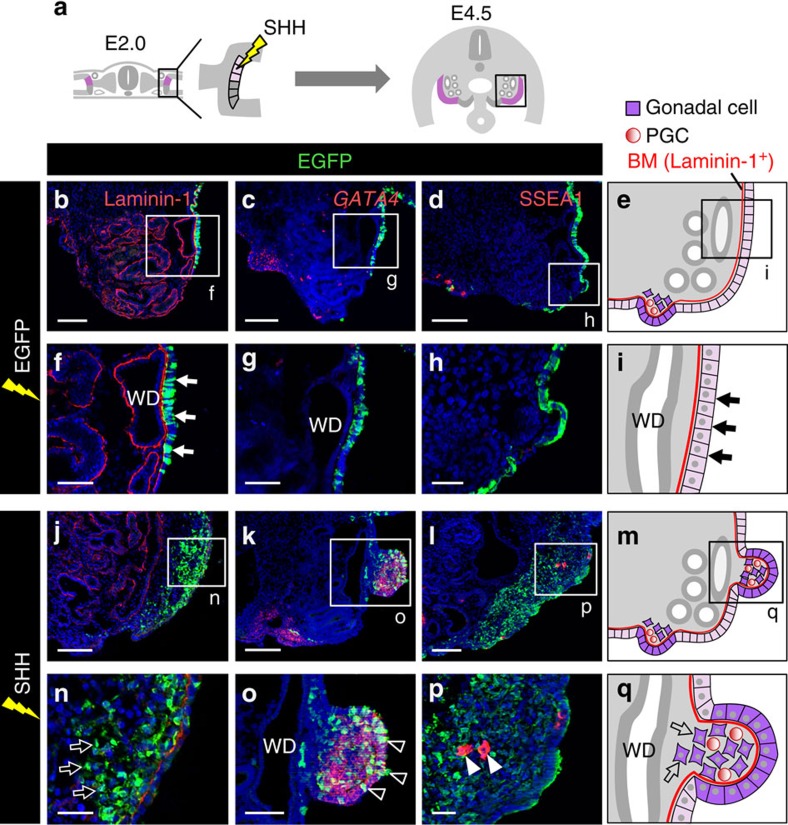
Ectopic gonadogenesis in MCPCs is triggered by SHH. (**a**) *SHH* expression plasmid was electroporated into MCPCs (pale pink) at E2.0. (**b**–**d**) Transverse views (corresponding to the boxed region in **a**) of the mesonephros in the E4.5 EGFP-electroporated control embryo. Images depict staining for laminin-1 protein (**b**), *GATA4* mRNA (**c**) or SSEA1 protein (**d**). (**e**) Illustrations of MCPC-derived cells after overexpression of control EGFP. (**f**–**i**) Magnified views of boxed region in **b**–**e**. The Wolffian duct (WD) is indicated. In control EGFP-electroporated embryos, MCPCs remained as epithelia (arrows in **f** and **i**) overlying a basement membrane (BM, laminin-1 positive) and did not express *GATA4* mRNA (**g**) or attract SSEA1-positive PGCs (**h**). (**j**–**l**) Boxed area in **a** in an *SHH*-transfected embryo, showing the distribution of laminin-1 (**j**), *GATA4* mRNA (**k**) or SSEA1-positive PGCs (**l**). (**m**) Illustrations of SHH-overexpressing MCPC-derived cells. (**n**–**q**) Magnified views of **j**–**m**. SHH overexpression caused MCPCs to undergo ingression (arrows in **n** and **q**), to express *GATA4* mRNA (**o** and **q**), often to begin forming a swell (arrowheads in **o**) and to retain ectopically localized SSEA1-positive PGCs (arrowheads in **p**). Scale bars, 100 μm (**b**–**d**,**j**–**l**); 25 μm (**f**,**g**,**o**); 50 μm (**h**,**n**,**p**).

We next investigated whether overexpressed SHH is capable of inducing gonadal development in MCPCs (Fig. 4a). As shown previously^29^, EGFP-electroporated (control) MCPCs remained as epithelia on a laminin-1-positive basement membrane at E4.5 (Fig. 4b,f, *n*=20). By contrast, the overexpression of SHH along with EGFP in MCPCs caused EGFP-positive cells to localize to the underlying stroma of the mesonephros. This observation indicates that, like GPCs, MCPCs undergo ingression if activated by SHH signalling (Fig. 4j,n; *n*=20). In addition, these MCPC-derived cells expressed the gonadal marker *GATA4* and formed a ridge similar to the gonadal primordium, whereas EGFP-treated (control) MCPCs did not (Fig. 4c,g, *n*=6; Fig. 4k,o, *n*=5; Fig. 4e,i,m,q).

LHX9 is strongly expressed in the overlying gonadal cortex and only weakly in the underlying mesenchyme of the gonad (Supplementary Fig. 9a–c). Likewise, the expression of LHX9 was observed in the ectopic gonad-like structure derived from MCPCs stimulated by overexpressed SHH (Supplementary Fig. 9d–j). Furthermore, PGCs localized to the mesonephros in addition to the gonad, and were surrounded by EGFP-positive cells derived from MCPCs, whereas PGCs did not localize to the mesonephros of control embryos (Fig. 4d,h, *n*=6; Fig. 4l,p, *n*=5; Supplementary Fig. 8). These results indicate that SHH signalling can trigger ectopic gonadogenesis in MCPCs by inducing ingression and well-organized differentiation into gonadal cells that can attract and retain PGCs (Fig. 4m,q). SHH appeared to activate its downstream signalling pathway specifically in early MCPC-derived cells. *PATCHED* was expressed in the MCPC-derived GFP+ cells stimulated by SHH at E3.5, but not E4.5. Furthermore, even in E3.5 embryos, Hedgehog signalling was not upregulated in the underlying mesonephric cells (Supplementary Fig. 10, *n*=5). Taken together, these results show that differences in Hedgehog signalling activity are responsible for the distinct behaviours of GPCs and MCPCs of E2.0 embryo, and that Hedgehog signalling orchestrates the onset of gonadogenesis in the GPCs.

The gonadal competence of the LPM in response to SHH appeared to depend on the proximodistal axis in E2.0 embryos. The lateral region of the LPM, neighbouring the MCPC, is called the somatopleural mesoderm, and it forms the body wall by undergoing ingression (Supplementary Fig. 11a)^13^. When SHH was overexpressed in the somatopleural mesoderm proximal to the MCPCs, these cells ectopically formed a well-organized genital ridge structure (in this structure, GATA4 was expressed broadly and LHX9 is expressed in the cortex; all of these properties were similar to those of the untreated gonad; Supplementary Fig. 11b–k, *n*=5). By contrast, the overexpression of SHH in somatopleural cells distal to MCPC did not form this structure (Supplementary Fig. 11q–u, *n*=5). Therefore, LPM cells appear to have different gonadal competence along the proximodistal axis at E2.0. Notably, when Hedgehog signalling was activated in MCPCs at E3.0 by SHH overexpression, they underwent neither ingression nor differentiation into gonadal cells (Supplementary Fig. 12, *n*=10). This observation suggests that MCPCs lose competence to differentiate into gonad at E3.0. Collectively, these findings indicate that the ability of LPM cells to respond to SHH signalling is spatiotemporally regulated.

### Regulation of BMP4 expression in the medial LPM

**Figure 5:**
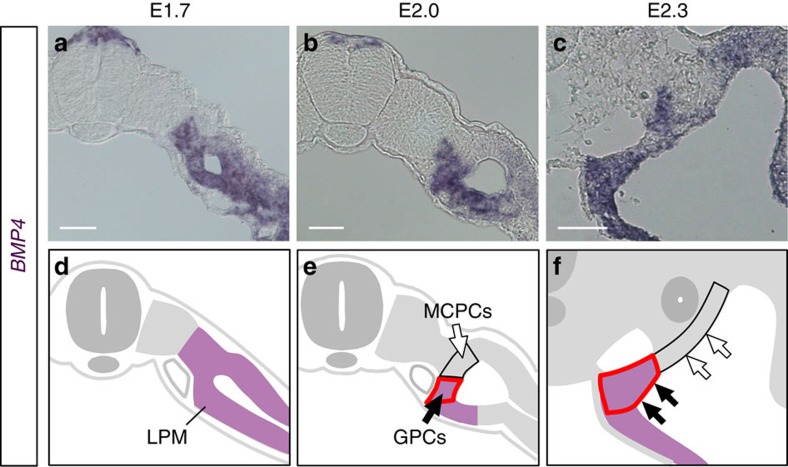
Transition of *BMP4* expression pattern in GPCs and MCPCs of E2 embryos. Transverse views of embryos at E1.7, E2.0 and E2.3 showing mRNA expression of *BMP4* (**a**–**c**); illustrations are shown in (**d**–**f**). BMP4 was expressed in the entire LPM of the E1.7 embryo (**d**). Expression disappeared in MCPCs (white arrows) but was maintained in the future gonadal area (outlined in red), including the gonadal coelomic epithelium (black arrows) and coelomic epithelial cell-derived underlying mesenchyme at E2.0 (**e**) and later (**f**). Scale bars, 50 μm.

We next investigated which events were induced in GPCs and MCPCs by Hedgehog signalling. BMP4, which acts downstream of Hedgehog signalling, regulates cell differentiation and migration in many processes, such as embryogenesis and cancer progression^24^,^38^. Furthermore, BMP4 is upregulated by SHH in the developing gut mesenchyme^26^,^39^. Therefore, we examined the expression of BMP4 in early chicken embryos. At E1.7, BMP4 is expressed throughout the LPM, including both GPCs and MCPCs, and thus establishes the mediolateral axis in the mesoderm (Fig. 5a,d)^25^. At E2.0, after gonadogenesis has been triggered, *BMP4* mRNA expression was downregulated in MCPCs, but maintained in GPCs (Fig. 5b,c,e,f). *BMP4* expression at E2.0 and thereafter was similar to that of *PATCHED* (Fig. 2h,i). These observations raised the possibility that differential expression of BMP4 is due to the differences in Hedgehog signalling activity in GPCs and MCPCs after E2.0.

**Figure 6:**
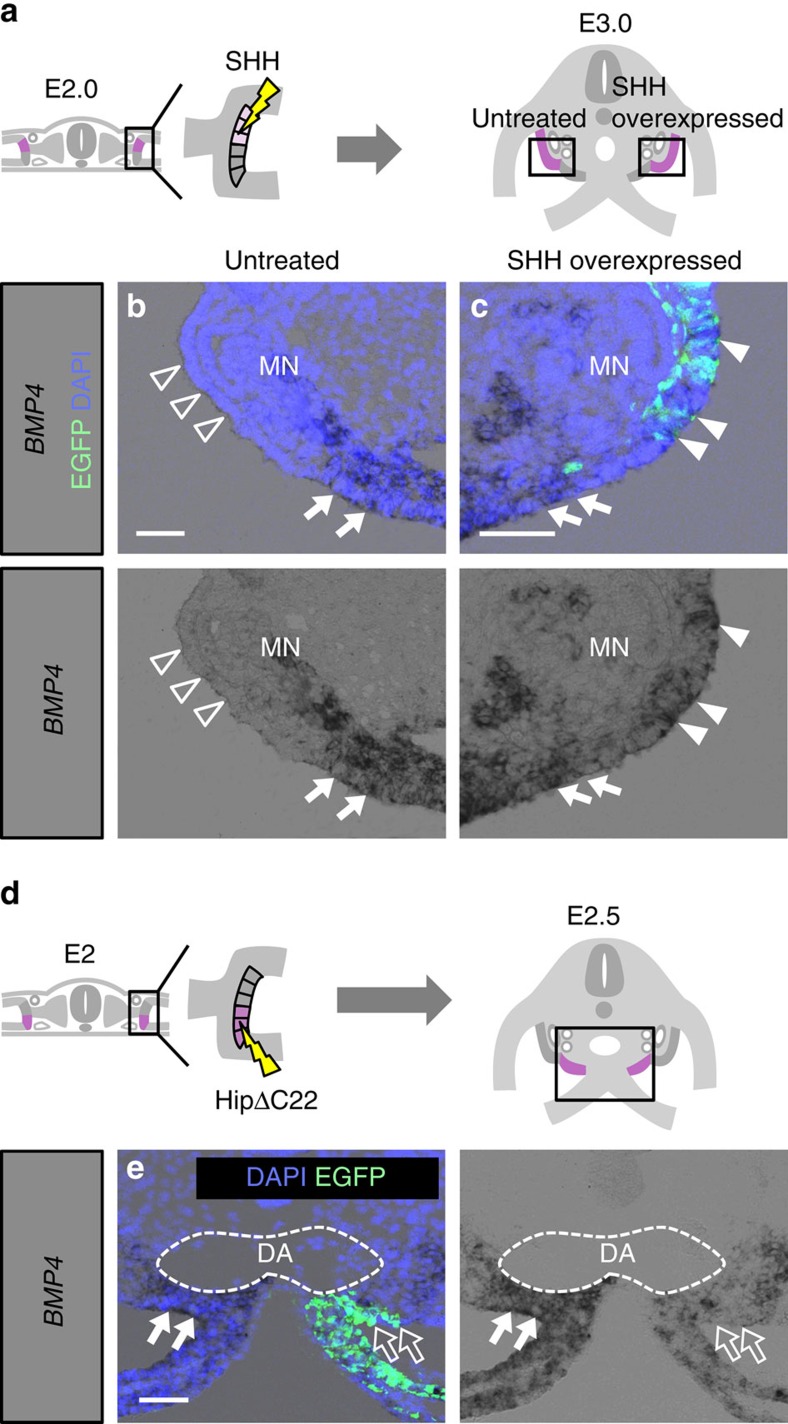
Differential *BMP4* expression in GPCs versus MCPCs induced by Hedgehog signalling. (**a**) *SHH* expression plasmid was electroporated into MCPCs at E2.0. (**b**,**c**) Transverse views of the mesonephros and presumptive gonad, corresponding to the boxed regions in **a**, at the untreated (control) side (**b**) or SHH-overexpressing side (**c**), stained for *BMP4* mRNA. *BMP4* expression was detected in the presumptive gonad (arrows in **b**) but not in the MCPCs (open arrowheads in **b**) on the untreated (control) side, but was detected in both MCPCs (white arrowheads in **c**) and GPCs (arrows in **c**) on the SHH-overexpressing side. (**d**) *Hip*Δ*C22* expression plasmid was electroporated into the GPCs in E2.0 embryos. (**e**) Transverse views of the presumptive gonadal region, corresponding to the boxed regions in **d**. *BMP4* expression was downregulated in the HipΔC22-electroporated side (open arrows) relative to the untreated (control) side (white arrows). MN: mesonephros; DA: dorsal aorta. Scale bars, 50 μm.

To address this question, we co-electroporated two plasmids, pCMV-SHH and pCAGGS-EGFP, into the MCPCs at E2.0 and investigated *BMP4* mRNA expression at E3.0 (Fig. 6a). BMP4 was expressed by GPC-derived cells, but not MCPCs, on the untreated side (Fig. 6b, *n*=6), whereas on the side with SHH overexpression, BMP4 was ectopically expressed in EGFP-positive MCPC-derived cells and their neighbours (Fig. 6c, *n*=6). When Hedgehog signalling was inhibited in GPCs by electroporation of HipΔC22 (Fig. 6d), *BMP4* expression was downregulated in GPCs on the electroporated side but not on the unelectroporated side (Fig. 6e, *n*=6). These results indicate that Hedgehog signalling is responsible for differential expression of BMP4 between GPCs and MCPCs after E2.0.

### MCPC and GPC behaviours are determined by BMP signalling

**Figure 7:**
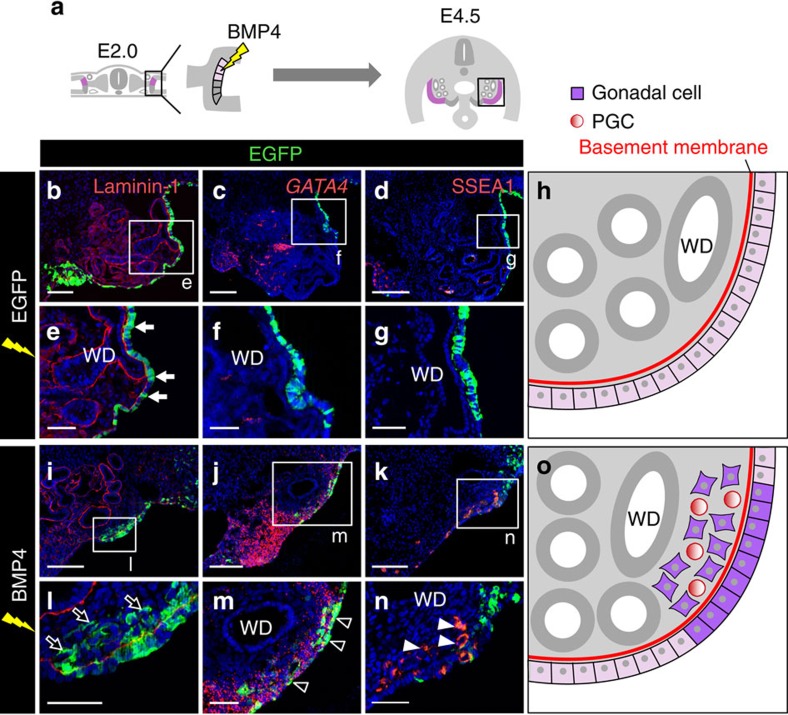
Gonadogenesis in MCPCs induced by BMP4. (**a**) *BMP4* cDNA was electroporated into MCPCs. (**b**–**d**) Transverse views of the E4.5 mesonephros after electroporation with control EGFP. Distributions of laminin-1, *GATA4* mRNA and SSEA1-positive PGCs are shown. (**e**–**g**) Magnified views of the boxed regions in **b**–**d**. MCPCs that remained as epithelia after electroporation with control EGFP (arrows in **b**). (**h**) Illustrations of control EGFP**-**overexpressing mesonephros. (**i–k**) Boxed region indicated in **a** in a BMP4-electroporated embryo. Laminin-1, *Gata4* mRNA and SSEA1-positive PGCs were detected. (**l**–**n**) Magnified views of the boxed regions in **i**–**k**. BMP4-overexpressing MCPC-derived cells were located in underlying kidney stroma (arrows in **l**), expressed *GATA4* mRNA (arrowheads in **m**) and induced ectopic localization of SSEA1-positive PGCs to their vicinity (arrowheads in **n**). (**o**) Illustrations of BMP4-overexpressing mesonephros. WD: Wolffian duct. Scale bars, 100 μm (**b**–**d**,**i**–**k**); 40 μm (**e**–**g**,**l**–**n**).

We next investigated whether BMP4 initiates gonadogenesis in MCPCs. To this end, pCAGGS-BMP4 and pCAGGS-EGFP were co-electroporated into MCPCs at E2.0 (Fig. 7a). As shown previously, the EGFP-electroporated (control) MCPCs remained as epithelia on the basement membrane (Fig. 7b,e, *n*=20; Fig. 7h)^29^ and neither expressed *GATA4* (Fig. 7c,f, *n*=5) nor attracted PGCs at E4.5 (Fig. 7d,g, *n*=6). By contrast, the overexpression of BMP4 initiated ingression of MCPCs to the underlying stroma (Fig. 7i,l, *n*=5; Fig. 7o). These MCPC-derived cells and their neighbours ectopically expressed *GATA4* and *LHX9*, although they did not form a genital ridge-like structure (Fig. 7j,m, *n*=5; Fig. 7o; Supplementary Fig. 9k–m, *n*=5). Furthermore, the BMP4-overexpressing MCPC-derived cells attracted PGCs to their vicinity (Fig. 7k,n, *n*=5; Fig. 7o; Supplementary Fig. 8). These results indicate that BMP signalling induces ingression of MCPC and differentiation into gonadal cells that can retain PGCs. Moreover, when BMP4 was overexpressed in the somatopleure proximal to MCPC, which formed an ectopic gonad on SHH overexpression, gonadogenesis was not complete: although these BMP4-overexpressing cells ectopically expressed *GATA4*, they did not express *LHX9* (Supplementary Fig. 11l–p, *n*=6).

**Figure 8:**
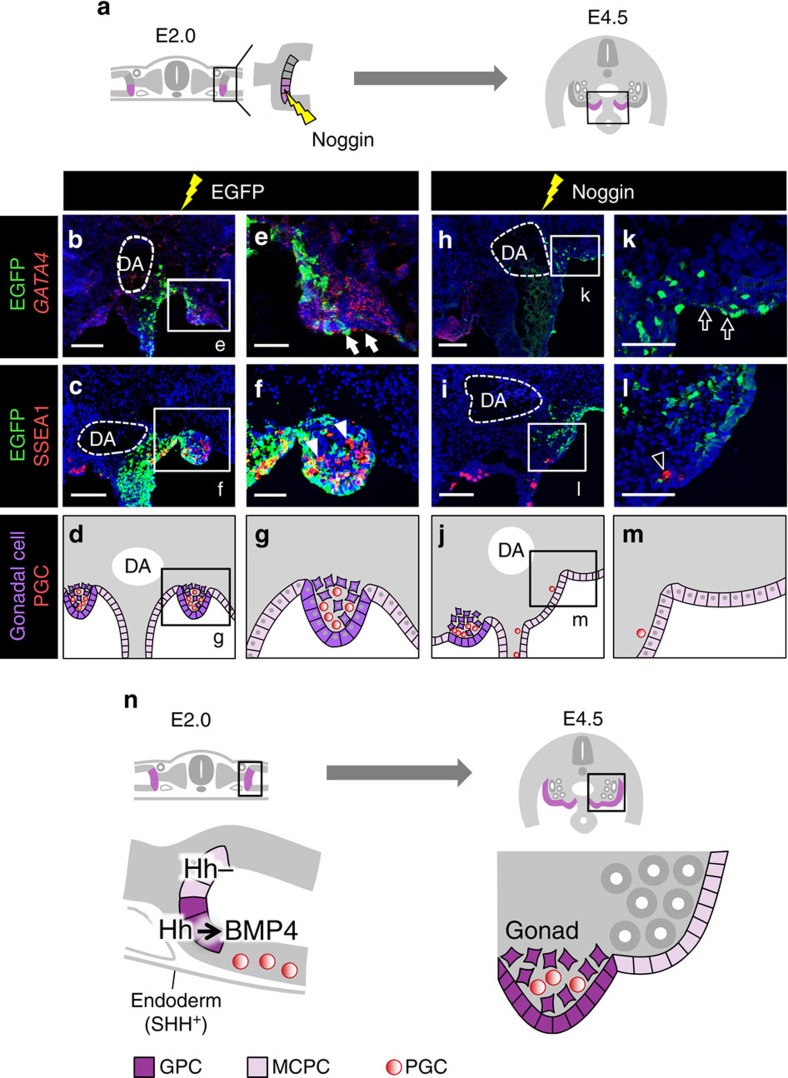
BMP signalling is necessary for GPCs to form gonad. (**a**) GPCs were transfected with *Noggin* expression plasmid. (**b**–**d**) Transverse views and a illustration, corresponding to the boxed region in **a** in a control EGFP-electroporated embryo. (**e**–**g**) Magnified views of the boxed regions in **b**–**d**. The gonad was observed as a swell that expressed GATA4 (arrows in **e**) and retained PGCs (arrowheads in **f**), as illustrated in **g**. (**h**–**j**) Transverse views of gonad in Noggin-overexpressing embryo. (**k**–**m**) Magnified views of the regions outlined in **h**–**j**. No GATA4-expressing swell formed at the presumptive gonadal region (arrows in **k**), and PGCs were scattered rather than gathered (arrowhead in **l**), as illustrated in **m**. (**n**) A scheme summarizing our findings. In E2.0 embryos, GPCs are localized at a site facing dorsal aorta (DA). Hedgehog signalling (Hh) is activated in GPCs but not MCPCs, and this Hedgehog signalling triggers gonadogenesis by causing GPCs to undergo ingression, inducing GATA4 expression and creating PGC niche activity, through BMP4 expression. SHH, a Hedgehog ligand, is expressed in endoderm, adjacent to the GPCs and more distant from the MCPCs. Scale bars, 100 μm (**b**,**c**,**h**,**i**); 50 μm (**e**,**f**,**k**,**l**).

We next investigated whether GPCs require BMP signalling to initiate gonadal differentiation. To this end, we electroporated the secretory BMP antagonist Noggin^25^,^40^ into GPCs and investigated whether the gonad was formed at E4.5 (Fig. 8a). We observed neither the expression of *GATA4* nor the formation of a genital ridge at the Noggin-overexpressing side (Fig. 8h,k, *n*=5; Fig. 8m). By contrast, EGFP-overexpressing (control) GPCs or untreated GPCs formed a *GATA4*-positive genital ridge (Fig. 8b,e, *n*=10; Fig. 8d,g; Fig. 8h, *n*=5; Fig. 8j).

SSEA1-positive PGCs localized to the genital ridge derived from EGFP-overexpressing (control) GPCs (Fig. 8c,f, *n*=10; Fig. 8g). By contrast, in Noggin-overexpressing embryos, PGCs did not localize to the presumptive gonadal region at the electroporated side. Instead, more PGCs were retained at the genital ridge on the untreated control side (Fig. 8i,l, *n*=10; Fig. 8m; Supplementary Fig. 8). Although Noggin inhibits BMP2 and BMP7 as well as BMP4 (ref. 41), *BMP2* and *BMP7* mRNAs were not detected in the GPCs or surrounding cells at E2.0 (Supplementary Fig. 13). Taken together, these findings indicate that altered *BMP4* expression at E2.0 established a dorsal (MCPCs) and ventral (GPCs) patterning in the medial LPM and orchestrates GPC behaviours to initiate gonadogenesis downstream of Hedgehog signalling.

## Discussion

In most multicellular organisms, the gonad is essential for the production of offspring. However, the molecular mechanism underlying the onset of gonadogenesis in vertebrates has remained largely unexplored, mainly because the location of GPCs within the LPM and the precise timing of the onset of gonadogenesis remained unknown. In this study, we demonstrated that GPCs are positioned at the ventromedial LPM, and that these cells initiate gonadogenesis by undergoing ingression in chicken 2-day (E2.0) embryos. Meanwhile, the dorsomedial LPM cells covering the mesonephros (MCPCs) maintain their epithelial integrity. These distinct behaviours of GPC and MCPC appear to be controlled by Hedgehog signalling. Subsequent to these events, Hedgehog signalling triggers gonadal differentiation by activating *BMP4* expression in GPCs (Fig. 8n).

The earliest morphogenetic event of gonadogenesis is considered to be the emergence of the epithelial thickening called the genital ridge, which is identifiable starting at E3.0 in chicken and E10.0 in mouse embryos^28^. Here we showed that GPCs start undergoing ingression as early as E2.0 in chicken embryos (Fig. 1). As far as we know, this is the earliest tissue/cellular sign of gonadogenesis reported to date.

This earliest event of gonadal development is most likely evoked by SHH emanated from endoderm. Moreover, it is noteworthy that SHH overexpression also caused multiple features normally observed during gonadal development, including formation of the genital ridge-like structure and collection of PGCs. Collectively, these data show that SHH is the most upstream molecule involved in triggering and orchestration of gonadogenesis in chicken.

In this study, we demonstrated that GATA4 and LHX9, which are gonadogenesis-related transcription factors in mouse^16^,^17^, are downstream targets of Hedgehog signalling in chicken. Moreover, we found that BMP4 regulates gonadal initiation downstream of Hedgehog signalling. In chicken, forced expression of BMP4 enabled MCPCs to show several aspects of gonadal initiation, but could not induce the formation of a genital ridge-like structure in and around MCPCs. These results imply that Hedgehog signalling controls downstream molecules in addition to BMP4 to form the ridge structure.

In addition, we demonstrated that expression patterns of BMP4 dynamically change before and after gonadal initiation. Before E2.0, *BMP4* is broadly expressed in LPM cells including MCPCs and GPCs, and form the LPM structure^25^. Subsequently, *BMP4* expression is restricted only to GPCs after E2.0. An appropriate transition of *BMP4* expression might be important for correct formation of the LPM and subsequent gonadal initiation. Future studies should seek to elucidate the mechanisms underlying this transition in *BMP4* expression, in which Hedgehog signalling plays an essential role.

We also found that ectopic gonadal induction by forced expression of SHH or BMP4 depended on the position within the LPM, as well as on developmental stage. In E2.0, MCPCs could respond to both SHH and BMP4, nearby proximal somatopleural cells could respond to SHH but not to BMP4, and distal somatopleural cells far from MCPCs could respond to neither. At E3.0, MCPCs no longer responded to SHH. Thus, gonadal competence seems to be regulated within LPM cells in a spatiotemporal manner. This regulation might contribute to placing gonads in the correct position or to the robustness of gonadal formation.

Although SHH and BMP4 play central roles in the initiation of gonadal differentiation in chicken embryos, these molecules have not been shown to be necessary in mice so far^42^,^43^. However, Shh and Bmp4 are expressed in mice embryo in a manner similar to the chicken^32^,^44^. Furthermore, primary cilia responsible for the distribution of secreted Hedgehog affect the gonadal length, and BMP signalling is required for correct localization of PGCs at the early genital ridge in mice^44^,^45^. Hedgehog and Bmp4 signalling might be involved in the initiation of gonadal differentiation in mouse embryos. It would be interesting to determine whether, and to what extent, gonadal initiation processes vary among amniotes.

## Methods

### Chicken embryos

Fertilized chicken eggs were commercially obtained from the Shiroyama Farm (Sagamihara, Japan). All animal experiments were conducted with the ethical approval of Kyoto University (No.H2620).

### DNA constructions

The pCMV-*SHH* was a gift from Dr T. Ogura (Tohoku University). The cDNAs for mouse *Hip*Δ*C22* and chicken *BMP4* and *Noggin* were PCR-amplified using the following primers: mouse *Hip*Δ*C22*, 5′-TTTACGCGTATGCTGAAGATGCTCTCGTT-3′ and 5′-TTTGCTAGCCTACCTGGTCACTCTGCGGAC-3′; chicken *BMP4*, 5′-AATTACGCGTATGATTCCTGGTAACCGAAT-3′ and 5′-ATCTGATATCAGCGGCACCCGCACCCCT-3′; chicken *Noggin*, 5′-AATTCTCGAgATGGATCATTCCCAGTGCCT-3′ and 5′-ATCTGATATCTAGCAGGAGCACTTGCACT-3′. The amplified fragments were digested with MluI–NheI, MluI–EcoRV or XhoI–EcoRV and subcloned into pCAGGS.

### DiI labelling

DiI (Invitrogen) was dissolved in ethanol (0.1%), heated at 45 °C for 3 min and diluted 1:10 with 0.3 M sucrose. This solution was injected into the coelom of the right side of E2.0 embryos using a glass capillary. Thereafter, the embryos were incubated for various time periods, killed, fixed overnight at 4 °C in PBS containing 4% paraformaldehyde (PFA) and sectioned by cryostat at 10-μm thick on Platinum Pro-coated glass slides (Matsunami). Images were obtained on an AxioPlanII microscope with the Apotome system (Carl Zeiss).

### Immunostaining

Laminin-1 was detected as follows. After pre-blocking with blocking solution (1% blocking reagent (Roche)/0.1 M Tris–HCl (pH 7.5), 0.15 M NaCl, 0.1% Tween 20 (TNT)) for 1 h at room temperature (RT), the sections were incubated at 4 °C overnight using an anti-laminin-1 antibody (mouse 3H11, DSHB, 1:400). After three washes in TNT, the specimens reacted with anti-mouse IgG-Alexa Fluor 568-conjugated goat antibody (Invitrogen) diluted 1:500 with blocking solution for 1 h at RT. The sections were washed three times in TNT and sealed with FluorSave reagent (Calbiochem). To detect SSEA1, atypical PKC or CVH, cryostat sections were treated with 3% H_2_O_2_ in TNT for 30 min, washed three times in TNT and preblocked. The specimens were reacted with a 1:300 dilution of anti-SSEA1 (mouse MC-480, DSHB), anti-PKCζ (rabbit sc-216; Santa Cruz Biotechnology) or anti-CVH (rat, see also Supplementary Method) antibodies, and subsequently reacted with a 1:300 dilution of horseradish peroxidase (HRP)-conjugated anti-mouse IgM (rat 1B4B1, Southern Biotech), HRP-conjugated anti-rabbit IgG (donkey, Amersham Bioscience) or HRP-conjugated anti-rat IgG (donkey, Abcam). After three washes in TNT, the specimens were reacted with Tyramide Signal Amplification (TSA) plus Cy3 (PerkinElmer) for 5 min at RT. The sections were then washed three times in TNT and sealed. Fluorescence images were obtained on an Axioplan 2 microscope with the Apotome system.

### Section *in situ* hybridization

After two washes in TNT, cryostat sections were treated with hybridization buffer (Ultra Hyb; Ambion) and incubated for 5 min at 65 °C. The hybridization was carried out overnight at 65 °C in hybridization buffer containing a DIG-labelled RNA probe (1 ng ml^−1^). The sections were rinsed and washed in wash solution 1 (50% formamide, 5 × SSC, pH 4.5, and 1% SDS) at 65 °C for 30 min, washed twice for 30 min each in wash solution 2 (50% formamide and 2 × SSC, pH 4.5) at 65 °C, and washed in a 1:1 mixture of wash solution 2 and TNT for 5 min at 65 °C, followed by three washes in TNT. The sections were then preblocked, followed by an overnight incubation at 4 °C in a blocking solution containing an anti-DIG-alkaline phosphatase-conjugated antibody (Roche). After three washes in TNT, the sections were processed in 100 mM Tris–HCl (pH 9.5), 100 mM NaCl, 50 mM MgCl_2_, 0.1% Tween 20 (NTMT)/2 mM levamisole. Alkaline phosphatase activity was visualized by incubating specimens in NTMT containing 0.07 mg ml^−1^ nitroblue tetrazolium chloride (Roche), 0.035 mg ml^−1^ 5-bromo-4-chloro-3-indolyl phosphate (Roche) and 2 mM levamisole. After the colour reaction was stopped by washing twice in TNT, the sections were mounted in FluorSave reagent with 4,6-diamidino-2-phenylindole. Images were obtained on an Axioskop 2 plus microscope (Carl Zeiss).

### *In ovo* electroporation

Expression plasmids were suspended in EB buffer (Qiagen) containing 2% Fast Green FCF (Nacalai Tesque) and 8% sucrose. The DNA solution was injected into the coelom of the right side of E2.0 or E3.0 embryos with a glass capillary. For transfection of the DNA into MCPC or GPC of E2.0 embryos, a minus electrode (tungsten) and plus electrode (platinum) were placed on the right and left side of the embryo, respectively. By contrast, to transfer genes into the somatopleure of E2.0 embryos or the MCPC of E3.0 embryos, the minus and plus electrodes were set at the lower and upper ends of the embryo, respectively. Thereafter, an electric pulse (E2.0 embryo: 75 V (0.05 ms ON/1 ms OFF) and five timed pulses of 20 V (25 ms ON/475 ms OFF); E3.0 embryo 75 V (0.05 ms ON/1 ms OFF) and five timed pulses of 25 V (25 ms ON/475 ms OFF)) was applied using a CUY21 EX (BEX).

### Probes

Chicken cDNA fragments for *SHH* and *PATCHED* were provided by Dr C. Tabin (Harvard University). Chicken *IHH* cDNA fragments were given by Dr T. Suzuki (Nagoya University). The cDNAs for chicken *BMP2*, *BMP4*, *BMP7*, *GATA4* and *LHX9* were obtained by PCR using the following primers: Chicken *BMP2*, 5′-GCCTCTCGAGATGGTTGCCGCCACCCGCTC-3′ and 5′-ATCTGATATCAGCGGCACCCGCAGCCCT-3′; Chicken *BMP4*, 5′-AATTACGCGTATGATTCCTGGTAACCGAAT-3′ and 5′-ATCTGATATCAGCGGCACCCGCACCCCT-3′; Chicken *BMP7*, 5′-GCCTCTCGAGATGCATTCCCAGAGCGTTCA-3′ and 5′-ATCTGATATCTAATGACAGCCGCATGCTC-3′;

Chicken *GATA4*, 5′-GCCTCTCGAGATGTACCAGAGCTTAGCCAT-3′ and 5′-ATCTGATATCTTATGCCGTTATGATGTCCC-3′; Chicken *LHX9*, 5′-GCCTCTCGAGATGCTTTTCCACGGGATCTC-3′ and 5′-ATCTGATATCTTAGAAAAGGTTCGTTAAGG-3′. The amplified fragments were digested by MluI–EcoRV or XhoI–EcoRV and subcloned into pBluescript or pCMS. Digoxigenin- or DNP-labelled RNA probes were prepared according to the manufacturer's instructions (Roche).

### Preparation of an anti-CVH antibody

A full-length *Cvh* cDNA fragment was subcloned in-frame into pGEX-5X3 (Pharmacia). The GST–CVH fusion protein was purified using a GST fusion system (Pharmacia) according to the manufacturer's instructions. The purified protein (300 mg) was injected into a rat. The resulting antiserum was purified by affinity chromatography using GST–CVH-conjugated agarose beads, and was used as an anti-CVH antibody.

### Double visualization of mRNA and EGFP protein

The double visualization of mRNA with fluorescent protein signals was performed as follows. Sections were treated with 3% H_2_O_2_ in methanol for 30 min, and then washed three times for 5 min each in TNT (0.1 mM Tris–HCl pH 7.5, 0.15 mM NaCl and 0.1% Tween 20). They were then incubated with hybridization buffer (Ultra hyb; Ambion) for 5 min at 65 °C. Hybridization was carried out with a DNP-labelled RNA probe (1 ng ml^−1^). The sections were washed with wash solution 1 (50% formamide, 5 × SSC, pH 4.5, and 1% SDS), wash solution 2 (50% formamide and × SSC, pH 4.5) at 65 °C, and a 1:1 mixture of wash solution 2 and TNT. After washing three times in TNT for 5 min each at RT, the specimens were preblocked with blocking solution (1% blocking reagent (Roche)/TNT) for 1 h at RT, followed by overnight incubation at 4 °C in the blocking solution containing 1:1,000 dilutions of an anti-DNP-*HRP*-conjugated antibody (PerkinElmer) and anti-GFP antibody (rabbit, Invitrogen). After three washes in TNT, the specimens were reacted with TSA plus Cy3 system for 10 min at RT. The reaction was terminated by washing three times in TNT. To visualize EGFP, the sections were incubated with an anti-rabbit IgG-Alexa 488-conjugated goat antibody (Invitrogen) diluted 1:500 in blocking solution, for 1 h at RT.

To detect mRNA with NBT/BCIP, sections were incubated with hybridization buffer for 5 min at 65 °C after two washes in TNT. The solution was replaced with prewarmed hybridization buffer containing Dig-labelled RNA probes and incubated overnight at 65 °C. The sections were washed with wash solution 1, wash solution 2, and a 1:1 mixture of wash solution 2 and TNT. After three washes in TNT, the samples were preblocked and incubated overnight at 4 °C in the blocking solution containing an anti-DIG-alkaline phosphatase-conjugated antibody and anti-GFP antibody. The samples were washed three times in TNT, followed by washing in NTMT (100 mM Tris–HCl, pH 9.5, 100 mM NaCl, 50 mM MgCl_2_ and 0.1% Tween 20)/2 mM Levamisole. The alkaline phosphatase activity was visualized by incubating the samples in NTMT containing nitroblue tetrazolium chloride, 5-bromo-4-chloro-3-indolyl phosphate and levamisole. The colour reaction was stopped by TNT washing, and then the sections were mounted. Images were obtained using a TCS SP6 microscope (Leica).

### Depletion of axial structures and explant culture

The surgical manipulations were performed on E2 embryos. A slit was made between the neural tube and right somite along the anteroposterior axis, with a sharpened tungsten needle. The embryo was then separated into a right axial structure-depleted side and a left control side. Both parts were fixed with PFA soon after the manipulation or incubated for 4 h at 38 °C on a 0.8-μm filter (Millipore) placed in a 6-cm Center-Well Culture Dish (Falcon) containing 10% fetal bovine serum/DMEM (Nissui).

### Visualization of CVH and EGFP proteins in whole E4.5 embryos

Double-fluorescent visualization of CVH and EGFP was performed as follows: E4.5 embryos were fixed with 4% PFA/PBS overnight at 4 °C, and then washed three times for 30 min each in 100 mM Tris–HCl (pH 7.5), 150 mM NaCl, 0.1% Tween 20, 0.1% TritonX (TNTT). The fixed embryos were treated with 2% blocking reagent (Roche)/TNTT for 60 min, incubated with 1/1,000 anti-CVH antibody (rat) and 1/1,000 anti-EGFP antibody (Clontech) in 2% blocking reagent (Roche)/TNTT overnight at 4 °C, and washed four times for 30 min each with TNTT. The embryos were then incubated with 1/500 Alexa Fluor 568-conjugated anti-rat antibody and 1/500 Alexa Fluor 488-conjugated anti-rabbit antibody (Invitrogen) diluted in 2% blocking reagent (Roche)/TNTT overnight at 4 °C, and washed four times for 30 min each in TNTT. To make samples transparent, they were finally treated with 75% glycerol/TNTT for 60 min. Images were obtained on an MVX10 microscope (Olympus).

### Data availability

Data supporting the findings of this study are available within the article and its Supplementary Information files and from the corresponding author on reasonable request.

## Additional information

**How to cite this article:** Yoshino, T. *et al*. Hedgehog–BMP signalling establishes dorsoventral patterning in lateral plate mesoderm to trigger gonadogenesis in chicken embryos. *Nat. Commun.* 7:12561 doi: 10.1038/ncomms12561 (2016).

## Supplementary Material

Supplementary Information Supplementary Figures 1-13
