# Supplementary material for: Hedgehog–BMP signalling establishes dorsoventral patterning in lateral plate mesoderm to trigger gonadogenesis in chicken embryos
Source: Nat Commun. 2016 Aug 25;7:12561. doi: 10.1038/ncomms12561 (PMC5007334; doi:10.1038/ncomms12561)
Supplement: Supplementary Information — Supplementary Figures 1-13 [file ncomms12561-s1.pdf]

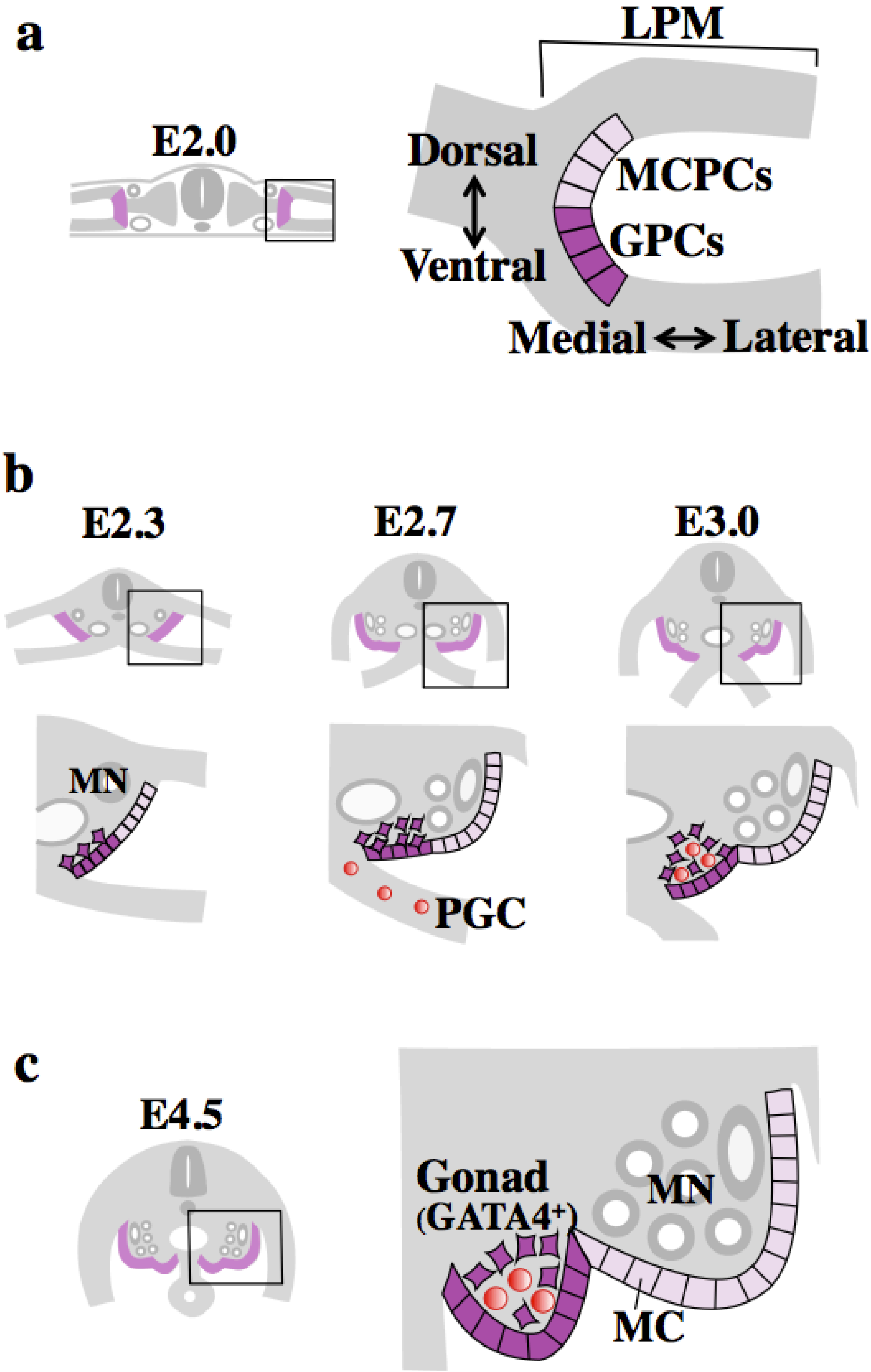

Supplementary Figure 1: Diagram of the localization of GPCs and MCPCs and their behavior in early chicken embryos

(a) Gonadal progenitor cells (GPCs: purple) are located at the ventral part of medial lateral plate mesoderm (LPM) of the E2.0 chicken embryo. By contrast, cells at dorsomedial LPM become mesonephric capsule progenitor cells (MCPCs: pale pink). **(b)** GPCs initiate gonadogenesis by undergoing ingression by E2.3. Thereafter, GPC-derived cells collect and retain primordial germ cells (PGCs), which come from mesentery before E2.7. Meanwhile, MCPCs remain as epithelia. **(c)** As a result of the different behaviors of GPCs and MCPCs, GATA4+ gonad and mesonephric capsule (MC) are formed appropriately by E4.5. MN: mesonephros.

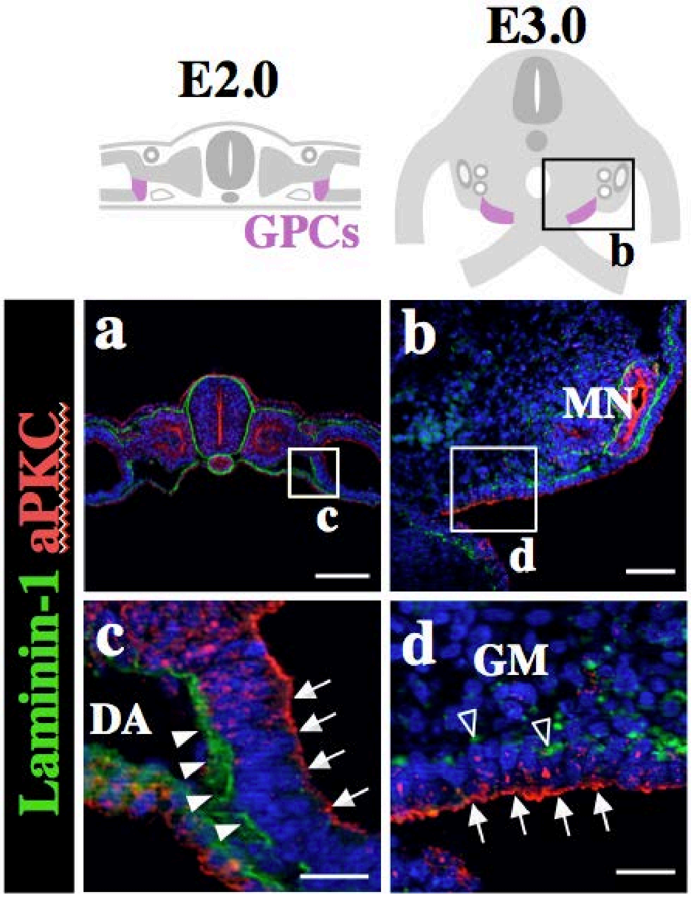

Supplementary Figure 2: Epithelial structure of GPCs at E2.0 and E3.0

(a, b) Immunostaining for atypical PKC (aPKC) and laminin-1. **(c, d)** Magnified views of the boxed regions in **a** and **b**. Basement membrane (laminin-1 positive) formed at the basal side of the GPCs in the E2.0 embryo (arrowheads in **c**). However, this structure was not maintained, and the laminin-1 protein was discontinuously distributed in the E3.0 embryo (arrowheads in **d**). In contrast, the apical surface established in the GPCs at E2.0 was maintained at E3.0 (arrows in **c, d**). DA: dorsal aorta; GM: gonadal mesenchyme; MN: mesonephros. Scale Bars: (**a**) 100 μm; (**b**) 50 μm; (**c, d**) 20 μm.

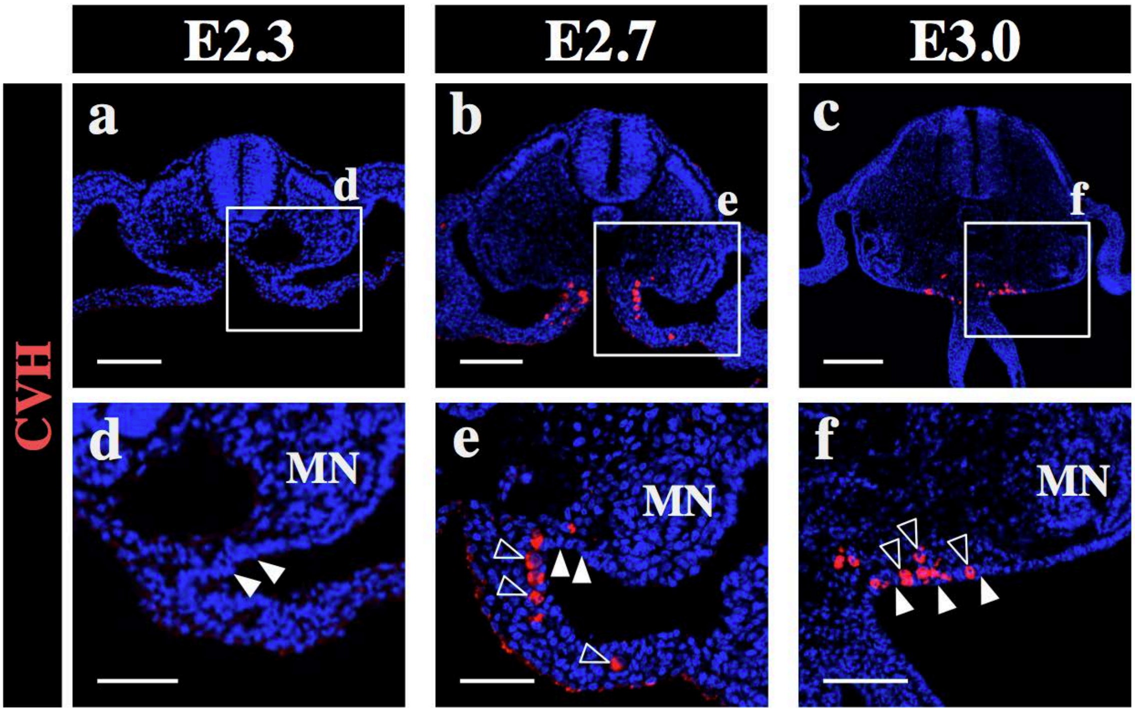

Supplementary Figure 3: PGC migration toward the presumptive gonadal region

(a–c) Immunofluorescence staining on transverse sections of E2.3-E3.0 embryos, for chicken vasa homologue (CVH: PGC marker). **(d–f)** Magnified views of the boxed regions in **a–c**. Filled arrowheads indicate the presumptive gonadal region. PGCs, which are produced in the extraembryonic region, had not reached the embryonic body at E2.3 (**d**) and appeared at the prospective gut mesenchyme at E2.7 (open arrowheads in **e**). The PGCs migrated and remained at the presumptive gonadal region in the E3.0 embryo (open arrowheads in **f**), and were absent from the mesonephros (MN). Scale Bars: (**a, b, f**) 100 μm; (**c**) 200 μm; (**d, e**) 50 μm.

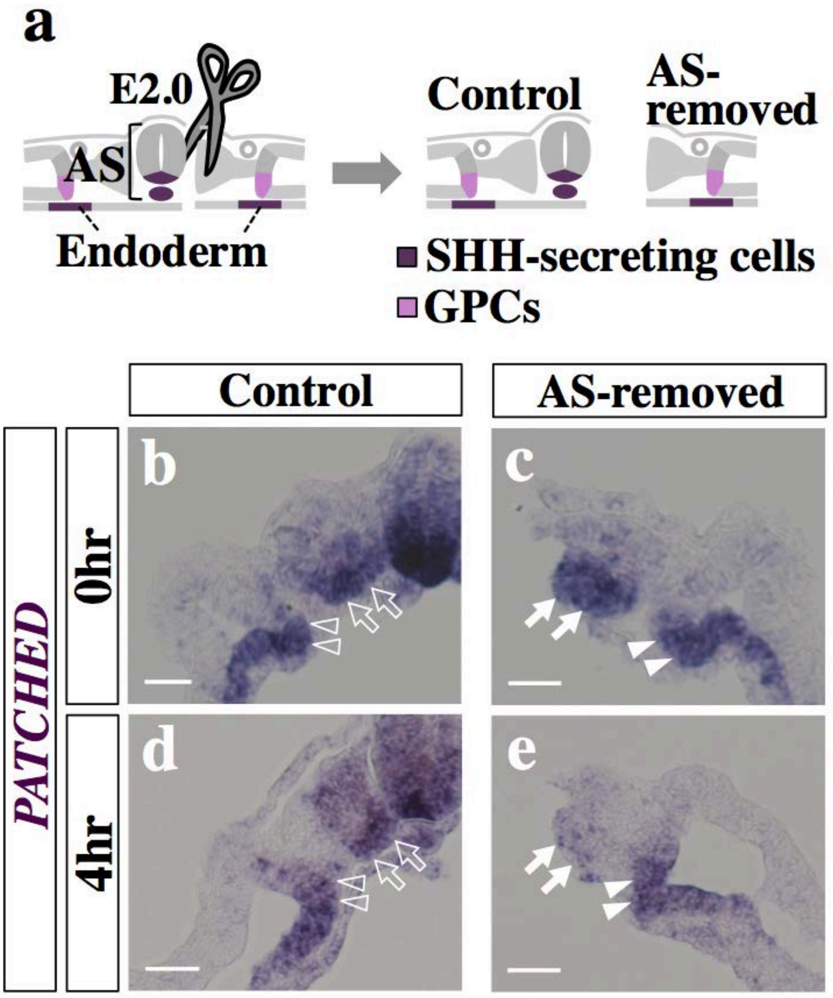

Supplementary Figure 4: Axial structures are dispensable for the Hedgehog signaling activation in GPCs

(a) SHH is secreted from axial structures (AS) as well as from the endoderm. E2.0 embryos were separated into an AS-retained part (control) and an AS-removed part. **(b–d)** Section *in situ* hybridization for *PATCHED* mRNA in the explants, which were fixed soon after manipulation (**b, c**) or fixed after a 4-hr incubation (**d, e**). *PATCHED* mRNA was expressed in the GPCs (arrowheads in **b** and **c**) and sclerotome (arrows in **b** and **c**) in the control and AS-removed sides soon after manipulation. Even after the incubation, the *PATCHED* expression was maintained in the GPCs on the AS-removed side as well

as on the control side (arrowheads in **d** and **e**), although its expression in the sclerotome was decreased on the AS-removed side compared with the control side (arrows in **d** and **e**). Scale bars: 50 μm.

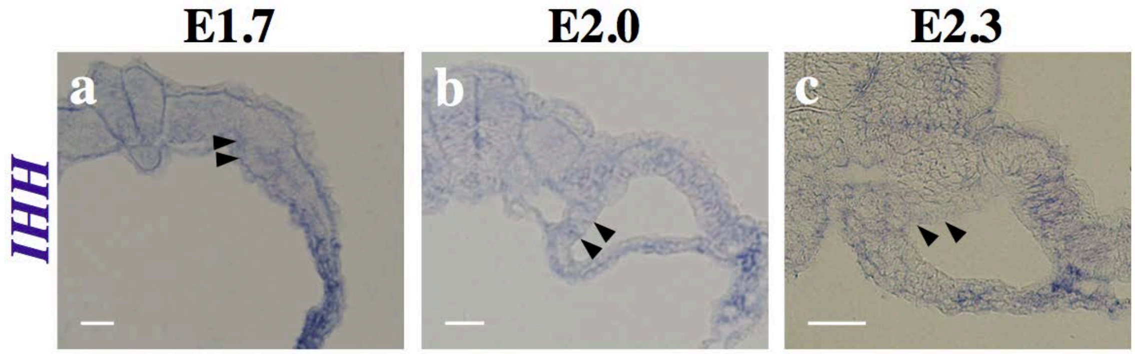

Supplementary Figure 5: Expression of *IHH* in embryos at E1.7, E2.0, and E2.3

(a–c) Transverse section *in situ* hybridization showing the expression of *IHH* mRNA in the E2 embryo. Arrowheads indicate the GPCs and future gonadal area. *IHH* was not expressed in the GPCs or its surrounding tissues. Scale bars: 50 μm.

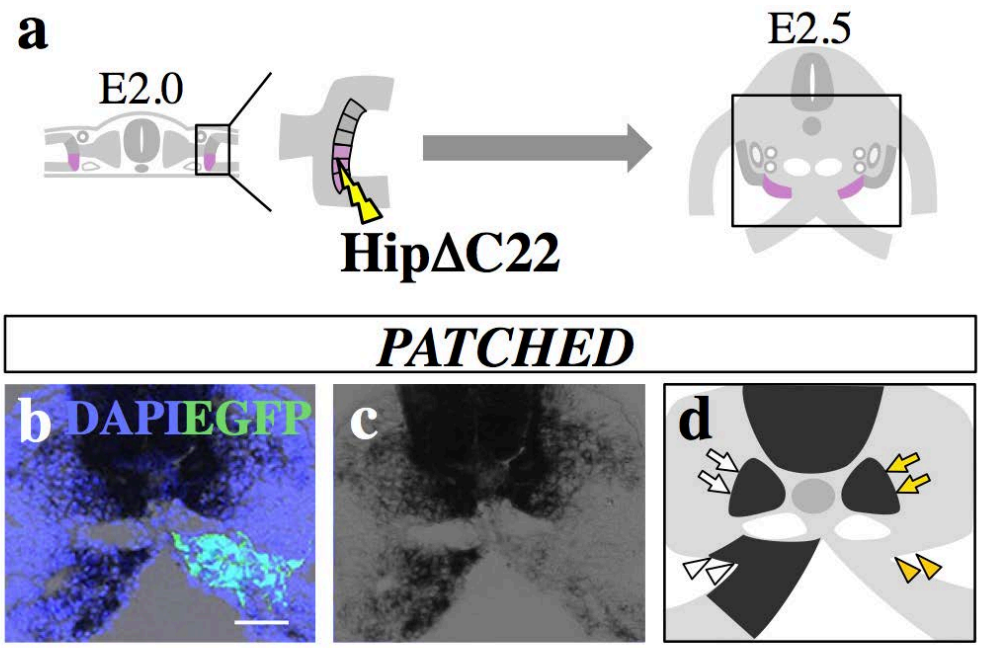

Supplementary Figure 6: HipΔC22 inhibits Hedgehog signaling specifically in the GPCs

(a) *HipΔC22* cDNA was electroporated with EGFP into GPCs (pink) at E2.0. The box corresponds to the area shown in **b-d**. **(b-d)** Section *in situ* hybridization for *PATCHED* mRNA, which was upregulated by Hedgehog signaling (**b, c**), and its illustration (**d**). The signals for *PATCHED* mRNA were much weaker in the GPCs on the HipΔC22-overexpressing side (yellow arrowheads) than on the control side (white arrowheads). In contrast, *PATCHED* mRNA was equally expressed in the sclerotome on the Hip-overexpressing side (yellow arrows) and the control side (white arrows). Scale Bars: 50 μm.

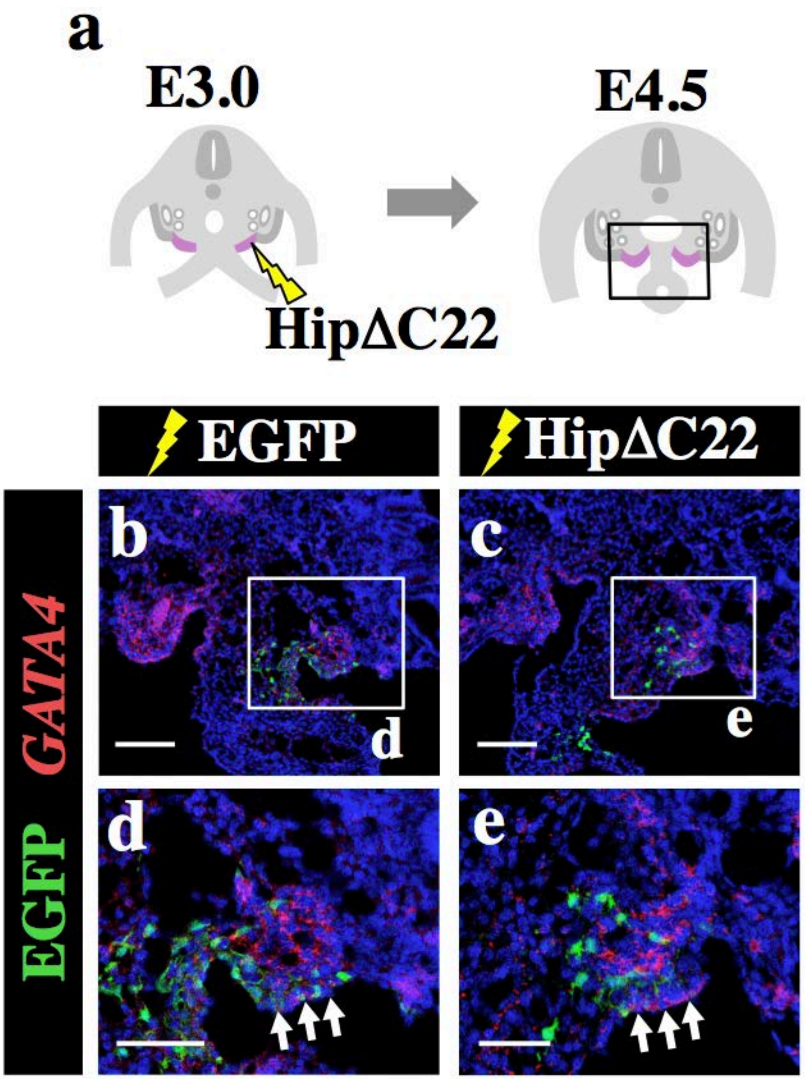

Supplementary Figure 7: Hedgehog signaling is dispensable for gonadogenesis from E3.0 to E4.5

(a) *HipΔC22* cDNA was electroporated along with EGFP into the GPCs (pink) at E3.0. **(b, c)** Boxed area in **a**, showing expression of *GATA4* mRNA after electroporation with (**b**) control EGFP or (**c**) HipΔC22. **(d, e)** Magnified views of the boxed region in **b** or **c** are shown. GPC-derived gonadal cells form the genital ridge, which express *GATA4*, in control EGFP-electroporated and *HipΔC22*-electroporated embryos (arrows in **d, e**). Scale bars: (**b**) 75 μm; (**c**) 100 μm; (**d, e**) 50 μm.

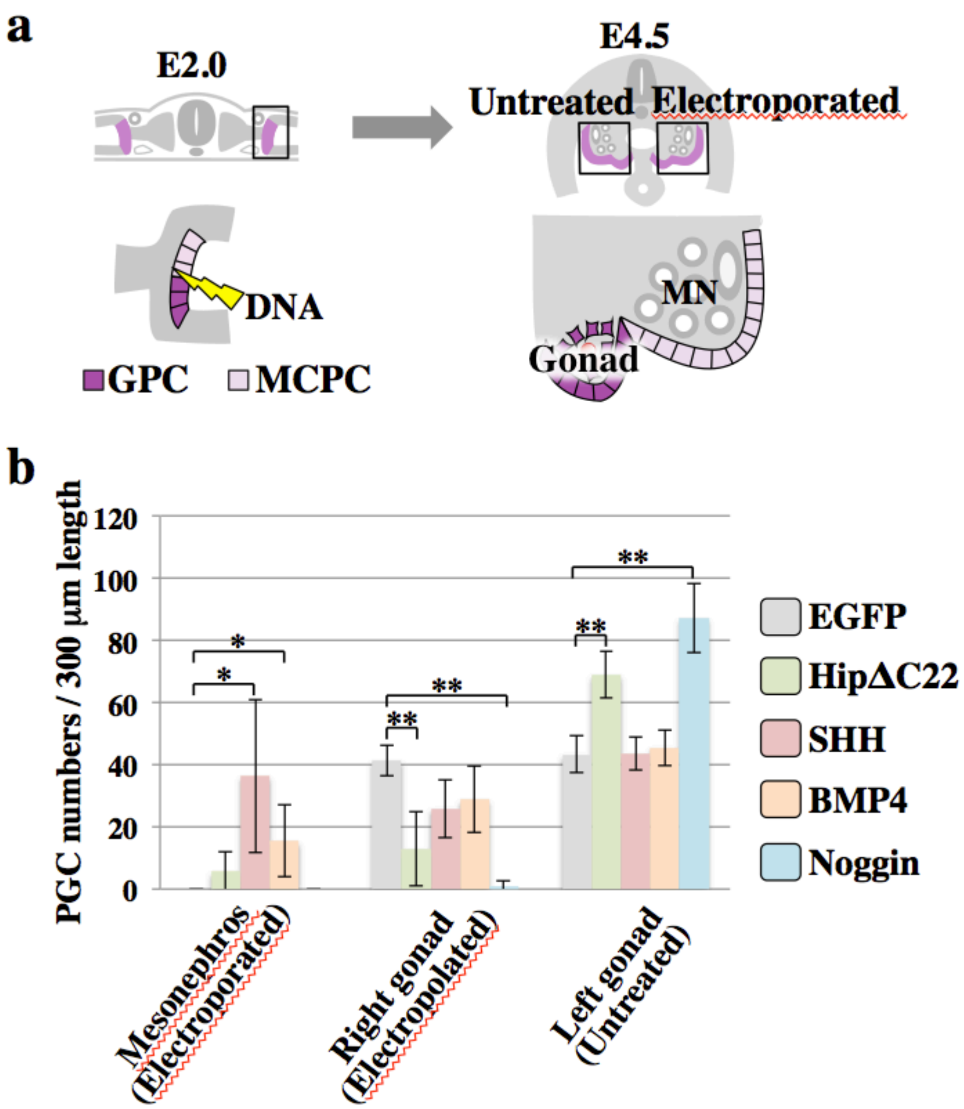

Supplementary Figure 8: Hedgehog and BMP signaling determines PGC localization in the urogenital system

(a) Ligand or inhibitor of Hedgehog or BMP signaling was electroporated into GPCs and MCPCs at E2.0. **(b)** Quantitative representation of PGC numbers in mesonephros (MN) and gonad per 300 µm length on the left (electroporated) side or right (control) side of E4.5 embryos. Urogenital systems from five embryos were assessed for each condition. Two group comparison was performed using the Student's *t*-test and $*P < 0.05$; $**P < 0.01$. Error bars represent SEM.

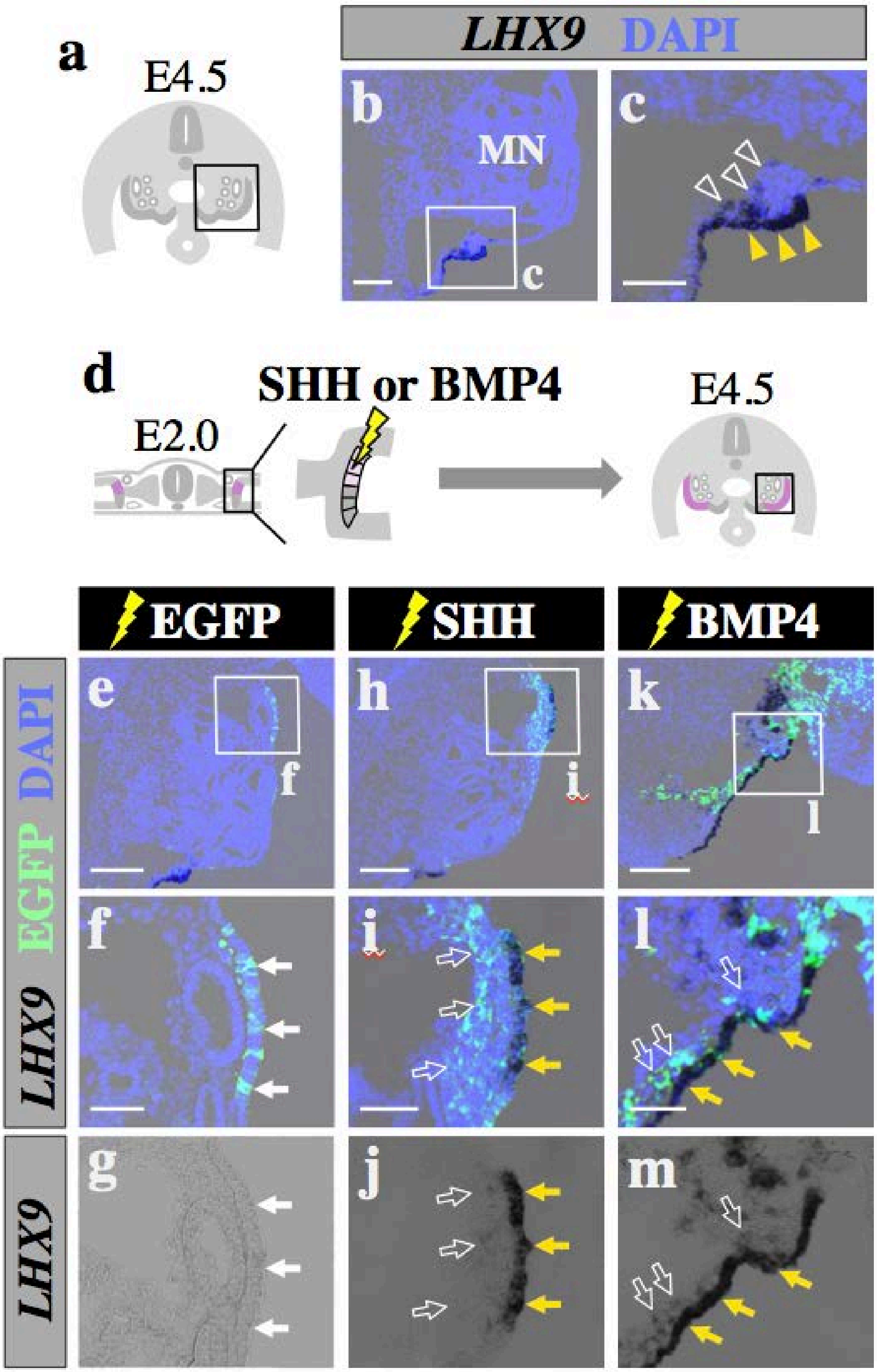

Supplementary Figure 9: SHH and BMP4 induce ectopic *LHX9* expression in MCPC derivatives in a similar manner as in the endogenous gonad

(a) Illustration of the transverse view of the E4.5 embryo. **(b)** The boxed area in **a**, showing the expression of *LHX9* mRNA. *LHX9* was specifically expressed in the gonad. **(c)** Magnified view of the boxed region in **b**. *LHX9* was intensely expressed in the gonadal cortex (yellow arrowheads) and weakly expressed in the underlying mesenchymal cells (open arrowheads), which arose from the GPCs. **(d)** Genes were electroporated into the MCPCs. **(e–m)** Transverse views of the E4.5 mesonephros and gonad in the boxed region in **d** (**e, h, k**), and magnified views of the boxed regions in **e, h,** and **k** (**f, i, l**). LHX9 was ectopically expressed in the overlying cortex (yellow arrows) but not in the underlying EGFP+ mesenchymal cells, which were derived from the MCPCs (open arrows) in the SHH- or BMP4-electroporated embryos. By contrast, no LHX9 expression was detected in the control EGFP-overexpressing MCPC-derived cells (white arrows). MN: mesonephros. Scale Bars: (**b, e, h, k**) 150 μm; (**c, f, i, j, l**) 50 μm.

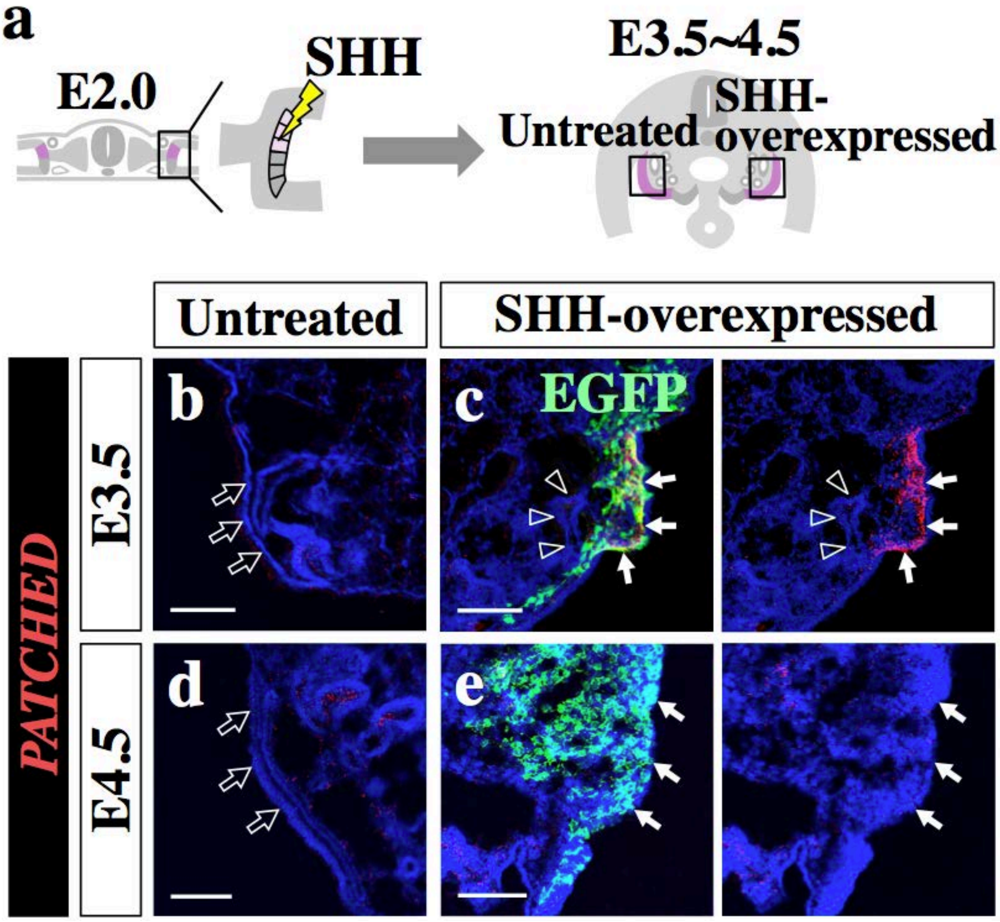

Supplementary Figure 10: Activation patterns of Hedgehog signaling that was established by overexpressing SHH in the MCPCs

(a) *SHH* cDNA was electroporated with EGFP into the MCPCs at E2.0. **(b–e)** Transverse views of the boxed region on the control untreated side (**b, d**) or the SHH-overexpressing side (**c, e**) in **a**, showing the activation pattern of Hedgehog signaling, which is indicated by the *PATCHED* mRNA expression. In E3.5 embryos, *PATCHED* mRNA was expressed in the MCPC-derived EGFP+ cells (white arrows in **c**) but not in cells of the underlying tissues such as the Wolffian duct (open arrowheads) on the SHH-overexpressing side. MCPCs on the untreated side did not express *PATCHED* mRNA (open arrows in **b**). In E4.5 embryos, *PATCHED* mRNA was not

detected in the EGFP+ MCPC-derived cells on the SHH-overexpressing side (white arrows in **e**) or in the cells on the untreated side (open arrows in **d**). Scale Bars: (**b, c**) 50 μm; (**d, e**) 100 μm.

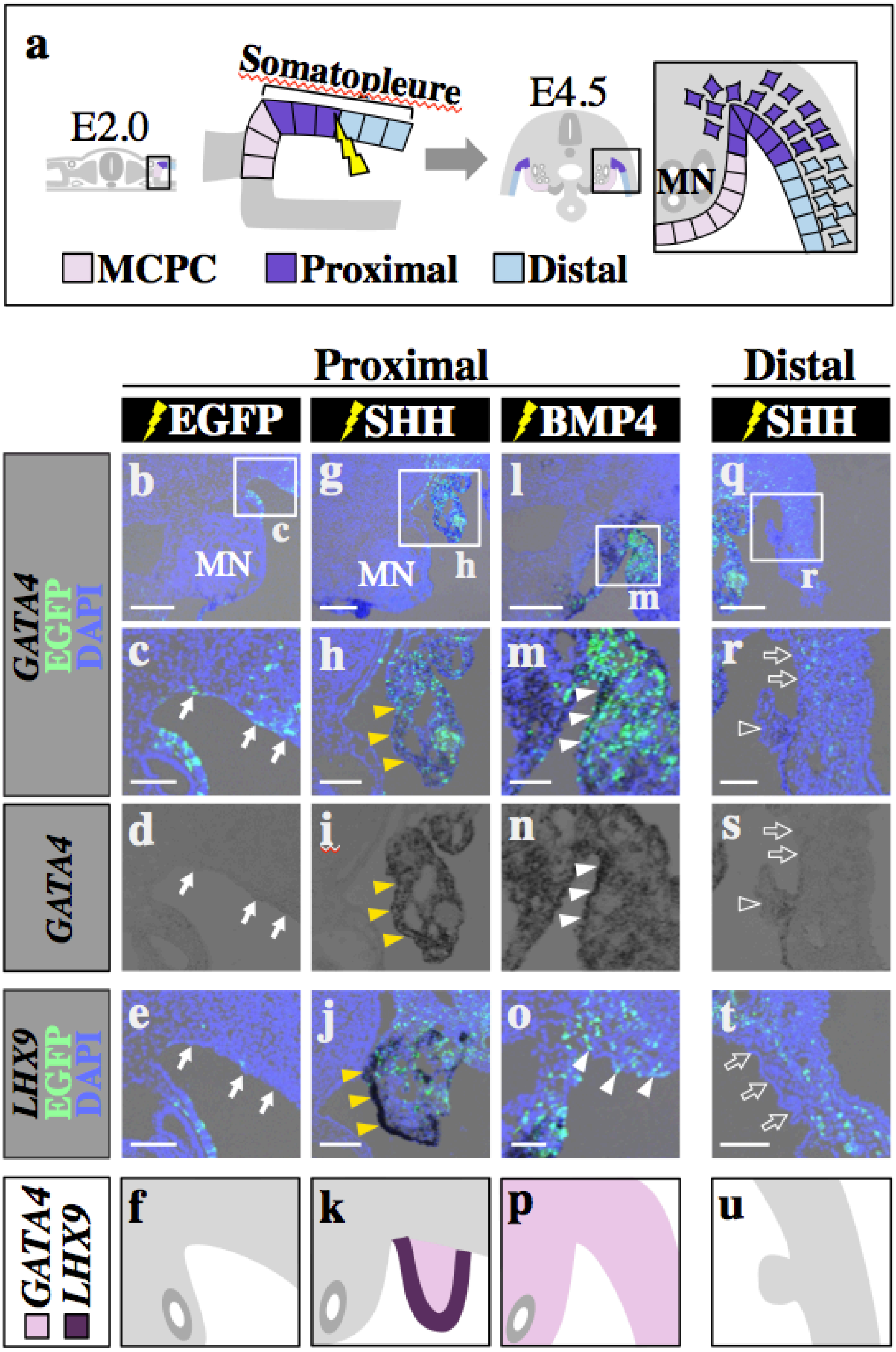

Supplementary Figure 11: LPM cells have different gonadal competences in response to SHH or BMP4 along the proximo-distal axis in the E2.0 embryo

(a) Somatopleural cells border the distal side of the MCPCs in the LPM and form body wall by undergoing ingression. Genes were electroporated into the proximal (dark blue) or distal (light blue) somatopleure at E2.0. **(b–u)** Transverse views of the mesonephros (MN) and somatopleure showing *GATA4* or *LHX9* mRNA expression in the E4.5 embryo. Magnified views corresponding to the boxes in **b, g, l, q** (**c, h, m, r**), Magnified photographs showing the expression of *LHX9* in the proximal somatopleure (**e, j, o**) and distal somatopleure (**t**) and their illustrations (**f, k, p, u**). SHH-transferred proximal somatopleural cells formed a ridge structure, which expressed *GATA4* broadly (yellow arrowheads in **h** and **i**) and *LHX9* in the cortex (yellow arrowheads in **j**), whereas the EGFP-transferred control cells did not form a ridge structure or express gonadal markers (white arrows in **c–e**). BMP4-overexpressing proximal somatopleural cells did not express *LHX9* (white arrowheads in **o**), although they expressed *GATA4* (white arrowheads in **m** and **n**). The expressions of *GATA4* and *LHX9* were not induced in the SHH-overexpressing distal somatopleure (open arrows in **r–t**), while a ridge structure was sometimes formed (open arrowheads in **r**). Scale Bars: (**b, g, q**) 200 μm; (**c, e, t**) 75 μm; (**h, j**) 100 μm (**l**) 150 μm. (**o, r**) 50 μm.

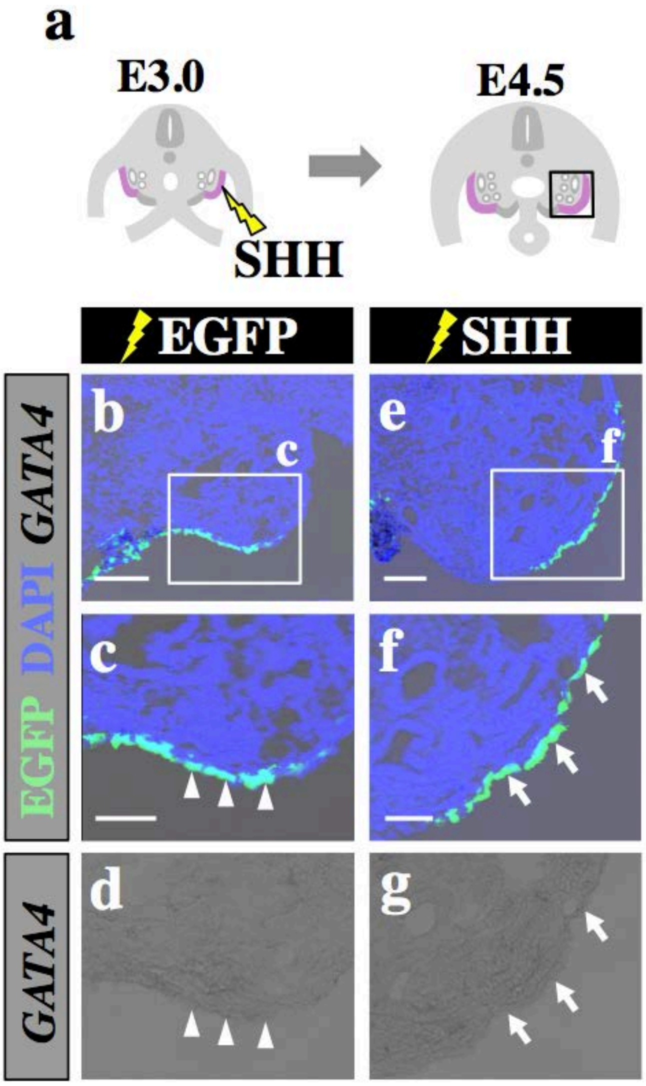

Supplementary Figure 12: MCPCs in the E3.0 embryo lack gonadal competence in response to SHH

(a) SHH was electroporated with EGFP into MCPCs (pink) at E3.0. **(b–g)** Transverse views of a mesonephric region corresponding to the boxed area of the E4.5 embryo in **a**, showing *GATA4* mRNA (**b, e**), and magnified views of the boxed region of **b** or **e** (**c, f**). SHH-overexpressing cells and EGFP-overexpressing control cells remained as epithelia and did not express *GATA4* mRNA (arrows in **f** and **g,** arrowheads in **c** and **d**). Scale Bars: (**b, e**) 100 μm; (**c, f**) 50 μm.

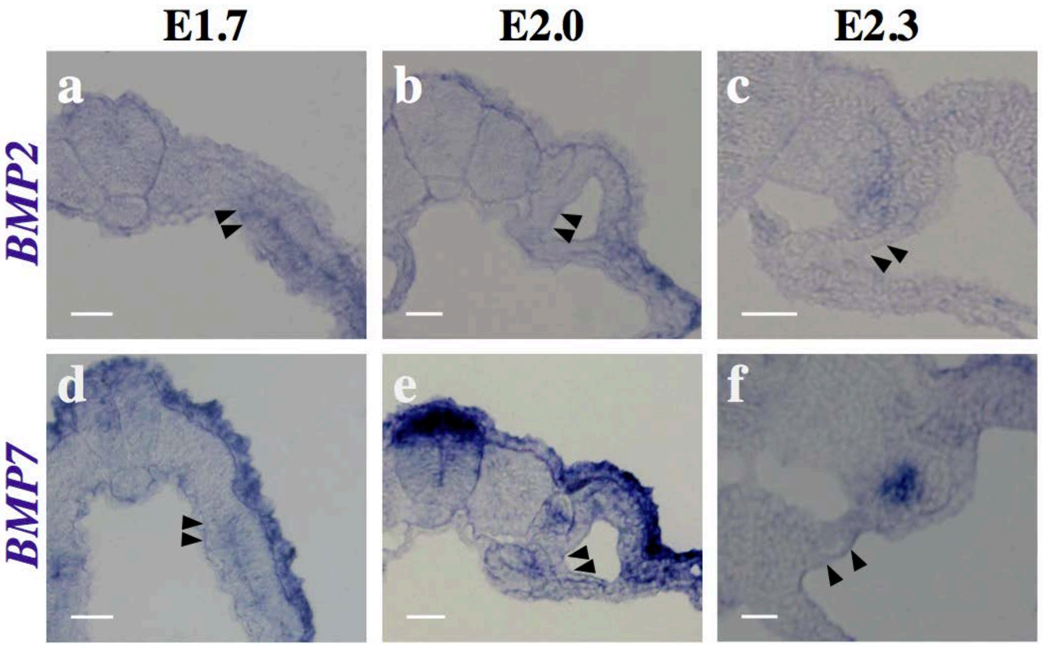

Supplementary Figure 13: Expression patterns of *BMP2* and *BMP7* in embryos at E1.7, E2.0, and E2.3

(a–f) Transverse section *in situ* hybridization for *BMP2* (**a–c**) or *BMP7* (**d–f**) in the E2 embryo. Arrowheads indicate the future gonadal area. *BMP2* mRNA was detected in the GPC at E1.7 (**a**) but not at E2.0 or E2.3 (**b, c**). BMP7 was not expressed in the GPC from E1.7 to E2.3 (**d–f**). Scale Bars: (**a–e**) 50 μm; (**f**) 25 μm.
